# Circadian clock control of MRTF/SRF pathway suppresses beige adipocyte thermogenic recruitment

**DOI:** 10.1093/jmcb/mjac079

**Published:** 2022-12-29

**Authors:** Xuekai Xiong, Weini Li, Ruya Liu, Pradip Saha, Vijay Yechoor, Ke Ma

**Affiliations:** Department of Diabetes Complications & Metabolism, Beckman Research Institute of City of Hope, Duarte, CA 91010, USA; Department of Diabetes Complications & Metabolism, Beckman Research Institute of City of Hope, Duarte, CA 91010, USA; Diabetes and Beta Cell Biology Center, Division of Endocrinology, Diabetes & Metabolism, Department of Medicine, University of Pittsburgh, Pittsburgh, PA 15213, USA; Department of Medicine, Baylor College of Medicine, Houston, TX 77030, USA; Diabetes and Beta Cell Biology Center, Division of Endocrinology, Diabetes & Metabolism, Department of Medicine, University of Pittsburgh, Pittsburgh, PA 15213, USA; Department of Diabetes Complications & Metabolism, Beckman Research Institute of City of Hope, Duarte, CA 91010, USA

**Keywords:** circadian rhythm, actin cytoskeleton, adipocyte differentiation, thermogenesis, energy balance, glucose metabolism, insulin sensitivity

## Abstract

The morphological transformation of adipogenic progenitors into mature adipocytes requires dissolution of actin cytoskeleton with loss of myocardin-related transcription factor (MRTF)/serum response factor (SRF) activity. Circadian clock confers temporal control in adipogenic differentiation, while the actin cytoskeleton–MRTF/SRF signaling transduces extracellular physical niche cues. Here, we define a novel circadian transcriptional control involved in actin cytoskeleton–MRTF/SRF signaling cascade that modulates beige fat thermogenic function. Key components of actin dynamic–MRTF/SRF pathway display circadian regulation in beige fat depot. The core clock regulator, brain and muscle arnt-like 1 (Bmal1), exerts direct transcriptional control of genes within the actin dynamic–MRTF/SRF cascade that impacts actin cytoskeleton organization and SRF activity. Employing beige fat-selective gene-targeting models together with pharmacological rescues, we further demonstrate that *Bmal1* inhibits beige adipogenesis and thermogenic capacity *in vivo* via the MRTF/SRF pathway. Selective ablation of *Bmal1* induces beigeing with improved glucose homeostasis, whereas its targeted overexpression attenuates thermogenic induction resulting in obesity. Collectively, our findings identify the clock–MRTF/SRF regulatory axis as an inhibitory mechanism of beige fat thermogenic recruitment with significant contribution to systemic metabolic homeostasis.

## Graphical Abstract

**Graphical Abstract:**
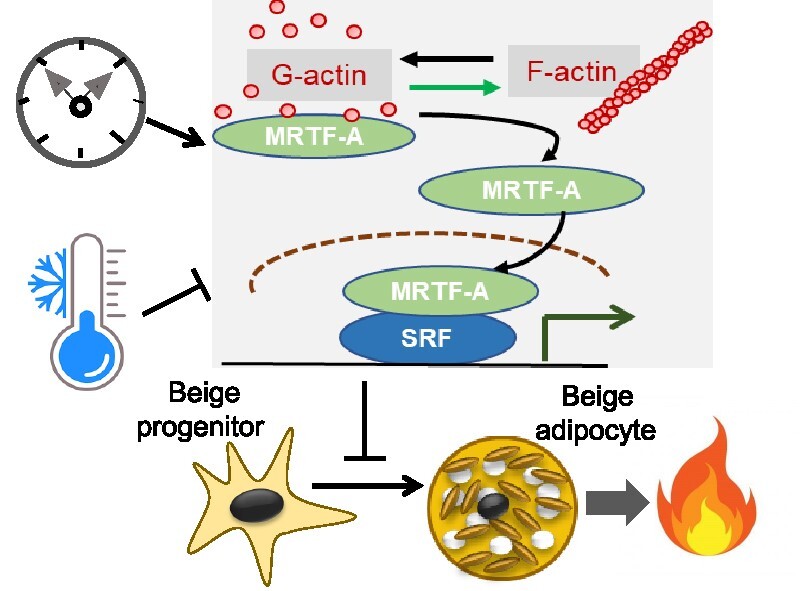

## Introduction

The development of mature adipocytes from adipogenic progenitors is characterized by drastic morphological transformation into a lipid-laden spherical shape from fibroblast-like morphology (Spiegelman and Farmer, 1982; Rodriguez Fernandez and Ben-Ze'ev, 1989; Kanzaki and Pessin, 2001). This process requires extensive intracellular actin cytoskeleton remodeling with dissolution of filamentous actin (F-actin) stress fibers that precedes lipid accumulation. Serum response factor (SRF) and its co-activator, myocardin-related transcription factor-A/B (MRTF-A/B, also known as MKL1/2), mediate the transcriptional response to actin dynamic that alters the ratio between monomeric globular actin (G-actin) and polymerized F-actin (Miralles et al., 2003; Olson and Nordheim, 2010). Besides its established role in driving distinct muscle lineage development (Wang et al., 2002, 2004; Li et al., 2005), progressive loss of MRTF/SRF activity is required for cytoskeleton reorganization that enables adipogenic induction during adipocyte formation (Spiegelman and Farmer, 1982; Liu et al., 2020). Recent studies indicate that MRTF/SRF suppresses mesenchymal precursor commitment to beige adipocyte lineage that possesses inducible thermogenic capacity, with loss of MRTF-A leading to browning of beige fat depot (McDonald et al., 2015; Rosenwald et al., 2017; Liu et al., 2020).

Functionally distinct adipose depots, including the visceral white, thermogenic brown, and beige adipose tissues, play specific roles in energy homeostasis (Wu et al., 2012; Sidossis and Kajimura, 2015; Cohen and Kajimura, 2021). While white adipocytes in visceral depots specialize in lipid storage, beige adipocytes residing in subcutaneous depots possess cold-inducible thermogenic activities for energy dissipation. This thermogenic property involves uncoupled mitochondrial respiration mediated by uncoupling protein 1 (UCP1) upon cold exposure or β-adrenergic stimulation. Exploring regulatory pathways to expand or activate the energy-dissipating capacity of beige fat may promote energy balance to protect against obesity (Boss and Farmer, 2012; Stanford et al., 2013). The recent discovery of the inhibitory effect of MRTF/SRF on beige adipogenesis suggests that this regulatory cascade could be targeted to augment beige fat capacity.

The circadian clock is a hierarchical system consisting of the central clock located in the suprachiasmatic nuclei and cell-autonomous peripheral clocks in nearly all tissues and cells (Takahashi, 2017; Finger et al., 2020). The central clock receives daily light signal transmitted from retina, which entrains the clock circuits in peripheral tissues under normal physiological conditions. A transcriptional negative feedback loop, coupled with translational and post-translational regulations, underlies the ∼24 h rhythmic oscillations in the central and peripheral clock circuits (Takahashi, 2017). Clock transcription activators, brain and muscle arnt-like 1 (Bmal1) and circadian locomotor output cycles kaput (CLOCK), form a heterodimer that activates the transcription of their repressors, the *Period* and *Cryptochrome* genes, with resultant Per/Cry-mediated repression of Bmal1/CLOCK constituting the negative feedback for clock oscillation. Rev-erbα and ROR-mediated *Bmal1* rhythmic expression re-enforces the core clock mechanism. Tissue-intrinsic clock circuits confer temporal control to metabolic pathways to orchestrate metabolic homeostasis (Panda et al., 2002), and their dysregulation predispose to the development of obesity and insulin resistance (Turek et al., 2005; Scheer et al., 2009; Xiong et al., 2021). Cell-autonomous peripheral clocks are present in adipose depots (Zvonic et al., 2006; Wu et al., 2007; Otway et al., 2009), and core clock components are involved in adipogenesis (Grimaldi et al., 2010; Guo et al., 2012). The essential core clock transcription activator Bmal1 inhibits differentiation of white and brown adipocytes (Guo et al., 2012; Nam et al., 2015b), while its repressor protein Rev-erbα (Nr1d1) promotes brown adipogenesis (Nam et al., 2015a).

In response to cell-surface cues, an intricate signaling cascade stimulates polymerization of monomeric G-actin to form F-actin that lowers effective actin monomer concentration (Miralles et al., 2003; Olson and Nordheim, 2010). Subsequent release of MRTF-A/B from sequestration by G-actin leads to its nuclear translocation to activate SRF-mediated transcription that drives fundamental cellular behaviors involving cytoskeleton, including adhesion, migration, and differentiation in developmental processes. Intriguingly, cyclic cues from serum stimulate actin remodeling in the liver and G-actin to F-actin ratio entrains clock, connecting actin dynamics with clock modulation (Gerber et al., 2013; Esnault et al., 2014). Whether circadian clock directly impacts actin cytoskeleton remains unknown, and mechanisms modulating MRTF/SRF activity besides actin dynamics is limited. In the current study, we identify that the actin cytoskeleton–MRTF/SRF signaling cascade is under direct transcriptional control of circadian clock, and this mechanism underlies clock modulation of beige fat thermogenic capacity.

## Results

### Bmal1 modulates actin cytoskeleton organization and exerts transcriptional control of actin–MRFT/SRF signaling pathway

**Figure 1:**
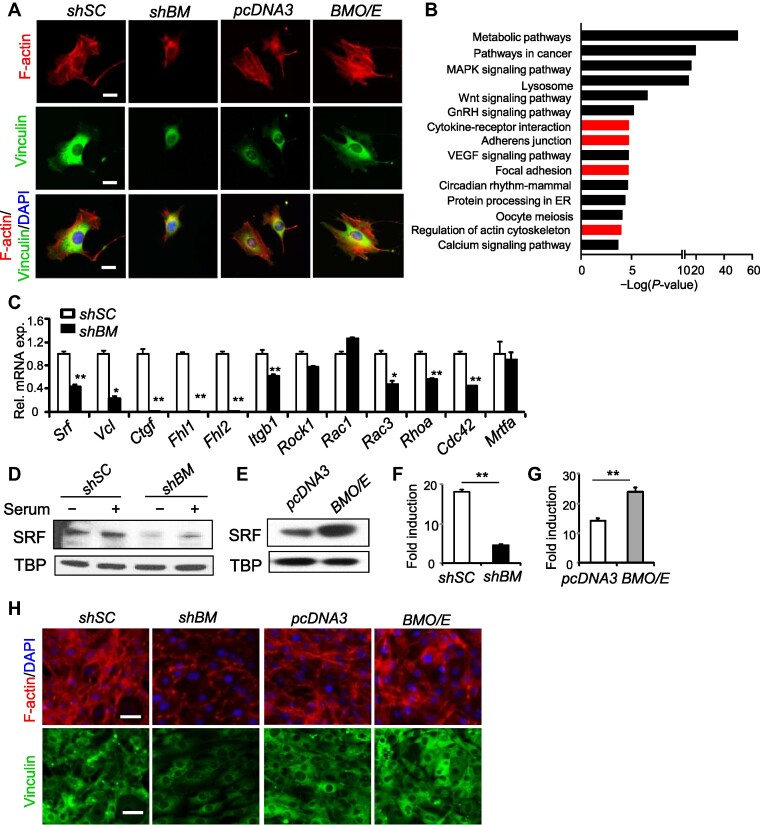
Bmal1 regulates the actin cytoskeleton and MRTF/SRF signaling pathway. (**A**) Representative confocal images of F-actin staining by phalloidin (red) and vinculin immunostaining (green) in C3H10T1/2 cells containing stable expression of shRNA of scrambled control (*shSC*) and Bmal1 (*shBM*), or *pcDNA3* and Bmal1 cDNA (*BMO/E)*. Scale bar, 25 μm. (**B**) KEGG analysis of enriched pathways of differentially expressed genes in *shBM* vs. *shSC* C3H10T1/2 cells (*n* = 3). Pathways related to actin cytoskeleton are highlighted in red. (**C**) RT-qPCR analysis of cytoskeleton and SRF-related genes in *shSC* or *shBM* C3H10T1/2 cells (*n* = 4). (**D** and **E**) Immunoblot analysis of SRF with or without serum stimulation in *shSC* and *shBM* (**D**) or *pcDNA3* and *BMO/E* cells (**E**). (**F** and **G**) Luciferase reporter assay of SRF activity in response to serum in *shSC* and *shBM* (**F**) or *pcDNA3* and *BMO/E* cells (**G**). Values were expressed as fold induction of serum stimulation over basal control without serum. **P ≤ 0.01 vs. respective controls (*n* = 4). (**H**) Representative images of F-actin and vinculin staining in *shSC* and *shBM*, or *pcDNA3* and *BMO/E* cells at Day 9 of C3H10T1/2 beige adipogenic differentiation. Scale bar, 100 μm.

C3H10T1/2 are mesenchymal stem cells capable of beige adipocyte lineage commitment (Tseng et al., 2008). In these mesenchymal beige precursors with loss- and gain-of-function of *Bmal1* (Nam et al., 2015b), we found striking changes in cell shape with altered actin cytoskeleton organization as shown by Phalloidin staining of F-actin (Figure 1A), with the quantification of F-actin staining cell area shown in Supplementary Figure S1A. Inhibition of *Bmal1* by stable shRNA silencing (*shBM*) reduced cell size and the abundance of polymerized F-actin. In contrast, *Bmal1* overexpression (*BMO/E*) augmented actin stress fibers with enlarged size. Global transcriptomics profiling by microarray in *shBM* and scramble control (*shSC)* C3H10T1/2 cells revealed, in addition to known clock-regulated metabolic and cancer-associated processes, enrichment of differentially regulated pathways related to cytoskeleton modulation, including focal adhesion, adherent junctions, actin cytoskeleton, and cytokine-receptor interaction (Figure 1B; Supplementary Figure S1B). Real-time quantitative polymerase chain reaction (RT-qPCR) analysis validated that various components involved in the cytoskeleton–MRTF/SRF signaling cascade were down-regulated with silencing of *Bmal1. Srf* transcript was reduced to ∼50% of *shSC* (Figure 1C). Expression of known MRTF/SRF target genes, *vinculin* (*Vcl*), *connective tissue growth factor* (*Ctgf* ), and *four-and-a-half-LIM-domain protein-1* (*Fhl1*) and *Fhl2*, were markedly decreased, suggesting attenuated SRF activity. SRF protein was reduced in cells with *Bmal1* silencing in the presence or absence of serum stimulation (Figure 1D), whereas overexpression elevated SRF protein level (Figure 1E). Analysis of SRF activity by an SRF response element (SRE)-driven luciferase reporter (SRE-Luc) revealed *Bmal1* modulation of SRF-mediated transcription, with *Bmal1* knockdown significantly attenuating (Figure 1F) and forced expression enhancing luciferase activity (Figure 1G). In *Bmal1*-deficient C3H10T1/2 cells at Day 9 of beige differentiation, actin filament was nearly lost with weak vinculin staining, whereas F-actin and vinculin-stained focal adhesions remained largely maintained in cells with *Bmal1* overexpression (Figure 1H). Prominent loss of F-actin organization and focal adhesion contact by vinculin staining was observed throughout the beige differentiation time course in *shBM* as compared to *shSC* controls (Supplementary Figure S1C and D).

**Figure 2:**
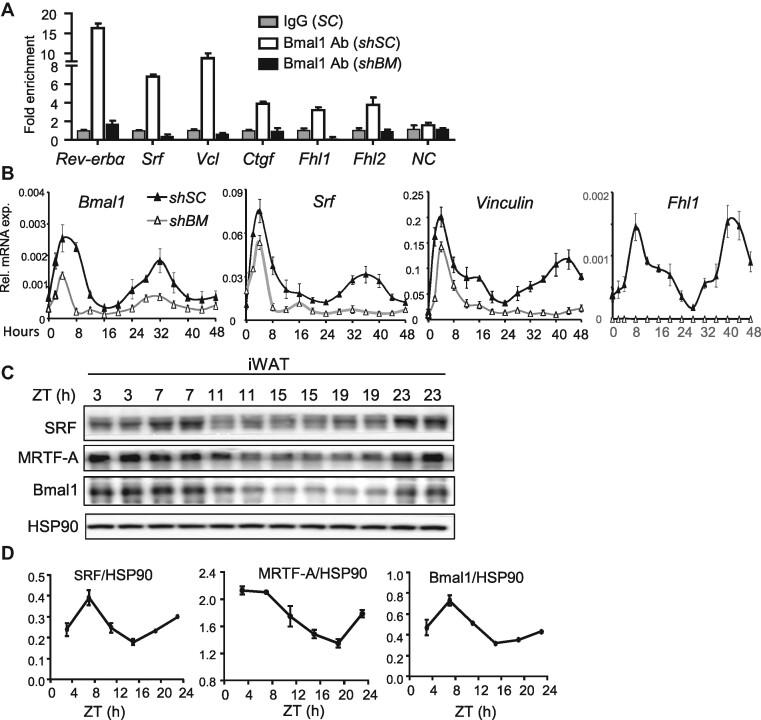
Bmal1 exerts direct transcriptional control of the cytoskeleton–MRTF/SRF pathway. (**A**) ChIP–qPCR analysis of Bmal1 chromatin occupancy on regulatory regions of MRTF/SRF pathway genes in *shSC* and *shBM* C3H10T1/2 cells. Values were calculated as percentages of input and normalized to IgG controls (*n* = 4). *NC*, negative control of *Rev-erb*α promoter upstream primers. (**B**) RT-qPCR analysis of circadian expression induced by serum shock every 4 h for 48 h in *shSC* and *shBM* C3H10T1/2 cells (*n* = 3). (**C** and **D**) Immunoblot analysis (**C**) with quantitation (**D**) of circadian expression of SRF and MRTF-A protein in iWAT of 10- to 12-week-old female mice for 24 h. Each time point represents a pooled sample of *n* = 4–5 mice.

These findings led us to explore whether Bmal1 exerts direct transcriptional control of genes within the actin cytoskeleton–MRTF/SRF cascade. Screening for putative Bmal1-binding sites of E- or E’-box elements (Rey et al., 2011; Chatterjee et al., 2013) within *cis-*regulatory regions identified several candidate targets within this pathway. Chromatin immunoprecipitation (ChIP)–qPCR analysis revealed enriched Bmal1 chromatin occupancies of proximal promoters of genes involved in cytoskeleton–SRF regulation, including *Srf, Vcl, Ctgf, Fhl1*, and *Fhl2* (Figure 2A), although they were comparatively less robust than that of a *Rev-erb*α promoter E-box. This finding of SRF and its target genes under the direct transcriptional control of Bmal1 suggests potential cooperativity between SRF and circadian regulation of these genes in beige fat. In *Bmal1*-deficient C3H10T1/2 cells, Bmal1 occupancies were mostly abolished to a similar degree as IgG control. To determine whether Bmal1 transcriptional regulation confers circadian oscillation of identified targets in cytoskeleton dynamics, we performed serum shock synchronization (Chatterjee et al., 2013), with induction of *Bmal1* rhythm that was significantly dampened in *shBM* cells across two circadian cycles, as expected (Figure 2B). Similar serum shock-induced oscillatory profiles were observed in *Srf* and MRTF/SRF target genes, *Vcl* and *Fhl*, accompanied with attenuation by

*Bmal1* inhibition. In inguinal subcutaneous white adipose tissue (iWAT), the representative beige adipose depot, SRF, and MRTF-A protein displayed daily rhythmicity with peak levels detected at early morning time point, which was in phase with Bmal1 expression (Figure 2C and D). MRTF-B expression was largely undetectable in iWAT at the circadian time points examined (Supplementary Figure S2A and B). CLOCK protein peak was detected at zeitgeber time (ZT)11, which overlapped with high Bmal1 levels between ZT7 and ZT15 but with a ∼4-h delay in peak expression (Supplementary Figure S2C).

### Selective ablation of Bmal1 in beige adipose tissue promotes browning

**Figure 3:**
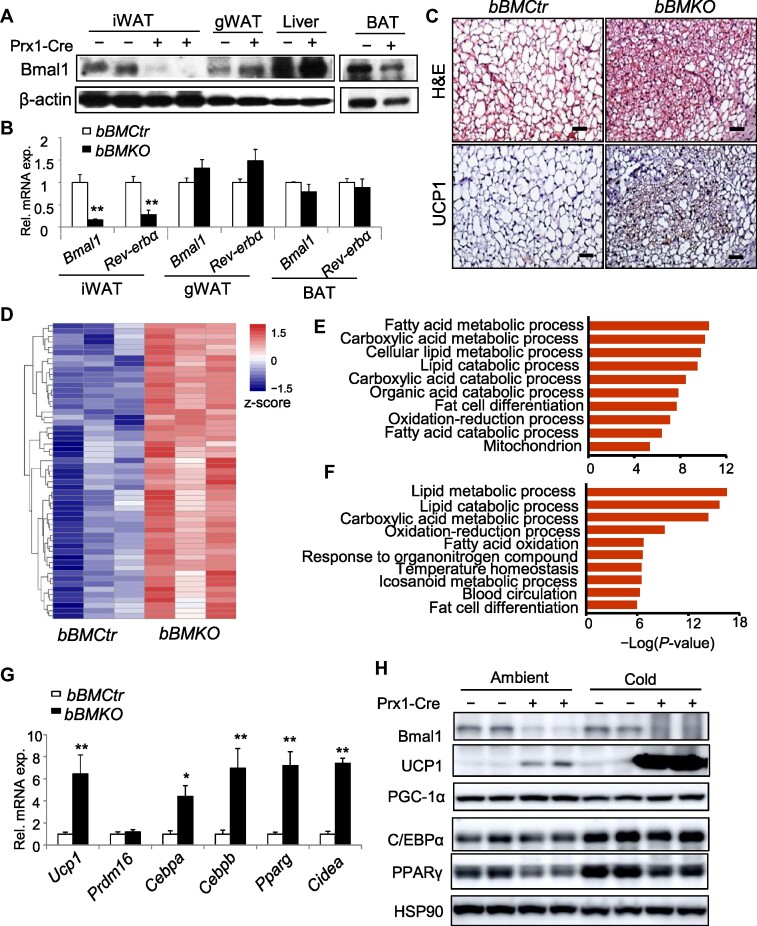
Selective ablation of *Bmal1* induces browning of beige fat. (**A** and **B**) Analysis of Bmal1 protein (**A**) and *Bmal1* mRNA (**B**) levels in fat depots and the liver of 8-week-old male *bBMCtr* and *bBMKO* mice at ZT3. (**C**) Representative images of H&E histology (upper panels) with UCP1 immunostaining (lower panels) of iWAT in 6-week-old male *bBMCtr* and *bBMKO* mice at ZT3. (**D**) Heatmap representation of up-regulated genes in mitochondria-related processes in iWAT from 6-week-old male *bBMKO* mice as compared to *bBMCtr* by RNA-seq analysis. (**E** and **F**) KEGG analysis of up-regulated pathways in *bBMKO* iWAT as compared to *bBMCtr* mice under ambient condition (**E**) and cold acclimation-induced pathways as compared to ambient condition in *bBMCtr* mice (**F**). *n* = 3/group. (**G**) RT-qPCR analysis of thermogenic and adipogenic genes in 6-week-old *bBMKO* and *bBMCtr* mice under ambient condition (*n* = 5/group). (**H**) Immunoblot analysis of thermogenic protein expression in iWAT of 8-week-old female *bBMCtr and bBMKO* mice under ambient or cold acclimation, with quantification shown in Supplementary Figure S3A. Each lane represents a pooled sample of *n* = 3–4 mice.

Prx1-Cre selectively targets subcutaneous beige adipose tissue progenitors and mature adipocytes without visceral white adipose or interscapular brown adipose tissue (BAT) involvement (Jeffery et al., 2014; Krueger et al., 2014; Sanchez-Gurmaches et al., 2015). To determine the role of the Bmal1–actin–MRTF/SRF regulatory axis in beige adipocyte development *in vivo*, we generated a mouse model with beige fat-selective *Bmal1* ablation using floxed *Bmal1* (BM^fl/fl^) and Prx1-Cre transgenic mice. In Prx1-Cre^+^/BM^fl/fl^ (*bBMKO*) mice, as compared to Prx1-Cre^–^/BM^fl/fl^ (*bBMCtr*) littermate controls, Bmal1 protein was absence in beige depot iWAT but not in gonadal/visceral classical white adipose tissue (gWAT), BAT, or the liver (Figure 3A). Loss of *Bmal1* transcript was corroborated in iWAT, together with attenuated expression of the Bmal1 target gene *Rev-erb*α (Figure 3B). Notably, relative to *bBMCtr*, iWAT of 6-week-old *bBMKO* mice displayed mitochondria-rich cytoplasmic staining and multi-locular lipid droplets indictive of beigeing (Figure 3C, upper panel), and UCP1 level was elevated as indicated by immunostaining (Figure 3C, lower panel). Consistent with the beigeing phenotype, RNA sequencing (RNA-seq) profiling revealed marked up-regulation of mitochondria-related processes in *bBMKO* iWAT (Figure 3D). Kyoto Encyclopedia of Genes and Genomes (KEGG) analysis identified that fatty acid and lipid metabolism were among the top enriched pathways (Figure 3E), and these processes overlapped with the metabolic signature induced by cold acclimation (Figure 3F), suggesting that the metabolic phenotype of *Bmal1-*deficient beige fat resembles cold-induced thermogenic induction. Indeed, thermogenic and adipogenic genes were induced in *bBMKO* iWAT, with ∼4- to 6-fold up-regulation of *Ucp1, Cebpb*, and *Ppar*γ (Figure 3G). UCP1 protein was markedly elevated in *Bmal1*-deficient iWAT under ambient temperature, with further induction by cold (Figure 3H; Supplementary Figure S3A). PGC-1α was moderately increased in *bBMKO*, while C/EBPα and PPARγ trended toward lower levels as compared to controls.

### Bmal1 deficiency in beige fat improves metabolic homeostasis

**Figure 4:**
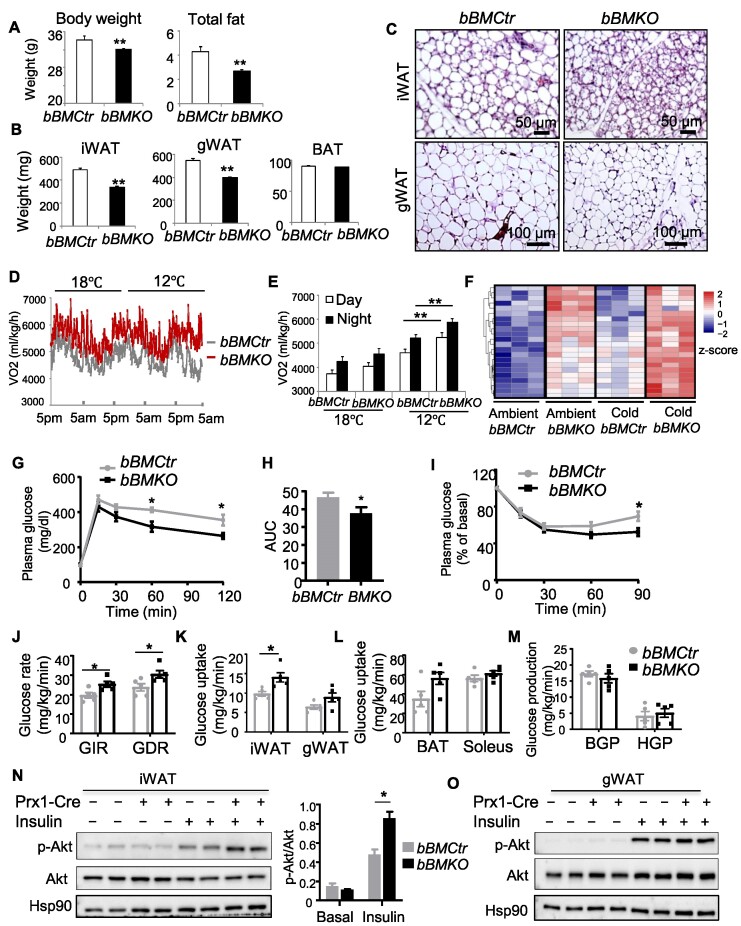
*Bmal1* deficiency in beige fat augments energy expenditure with improved glucose metabolism. (**A**–**C**) Total body weight and fat mass (**A**), fat tissue weight (**B**), and representative H&E histology of iWAT and gWAT (**C**) in 6-month-old male *bBMKO* and *bBMCtr* mice. *n* = 6–8/group. (**D** and **E**) Metabolic cage analysis of oxygen consumption rate (VO2) in male *bBMKO* and *bBMCtr* mice as shown by average tracing (**D**) and quantification (**E**) under ambient temperature and cold acclimation at 18°C or 12°C (*n* = 6/group). (**F**) Heatmap representation of up-regulation of fatty acid metabolic pathway by RNA-seq analysis in female *bBMKO* and *bBMCtr* mice under ambient or cold conditions (*n* = 3/group). (**G**–**I**) GTT with AUC quantification and ITT in 10- to 12-week-old male *bBMCtr* and *bBMKO* mice at ambient temperature (*n* = 6–7/group). (**J**–**M**) Hyperinsulinemic–euglycemic clamp analysis of steady-state GIR and GDR (**J**), glucose uptake in iWAT, gWAT, BAT, and soleus muscle (**K** and **L**), and BGP and HGP (**M**) from male *bBMKO* and *bBMCtr* mice (*n* = 5/group). (**N** and **O**) Akt phosphorylation in response to intraperitoneal 0.5 U/kg insulin stimulation in iWAT (**N**) and gWAT (**O**) in female *bBMKO* and *bBMCtr* mice. Each lane represents a pooled sample of *n* = 3 mice. *P ≤ 0.05, **P ≤ 0.01, by Student's *t*-test.

Given the metabolic benefit of inducible beige fat thermogenic activity, we next tested whether *Bmal1* ablation*-*induced beigeing is sufficient to improve energy balance. Loss of *Bmal1* in beige fat did not alter body weight or total fat mass in 8-week-old mice (Supplementary Figure S3B and C). By 6 months of age, however, *bBMKO* mice displayed significantly reduced body weight with ∼40% lower fat mass relative to littermate controls (Figure 4A). Both iWAT and gWAT were decreased, but not BAT (Figure 4B). *Bmal1*-deficient iWAT maintained enhanced beige characteristics at this age as compared to age-matched *bBMCtr* with smaller adipocytes in iWAT and gWAT (Figure 4C), suggesting cumulative effects of beigeing on energy balance with beige-selective *Bmal1* ablation. *bBMKO* mice at ambient temperature displayed a tendency toward higher oxygen consumption, which became significantly elevated compared to controls following cold acclimation at 12°C (Figure 4D and E). Marked inductions of lipid catabolic processes in *Bmal1*-deficient iWAT at ambient temperature with stimulation by cold from RNA-seq analysis further corroborated these findings (Figure 4F).

**Figure 5:**
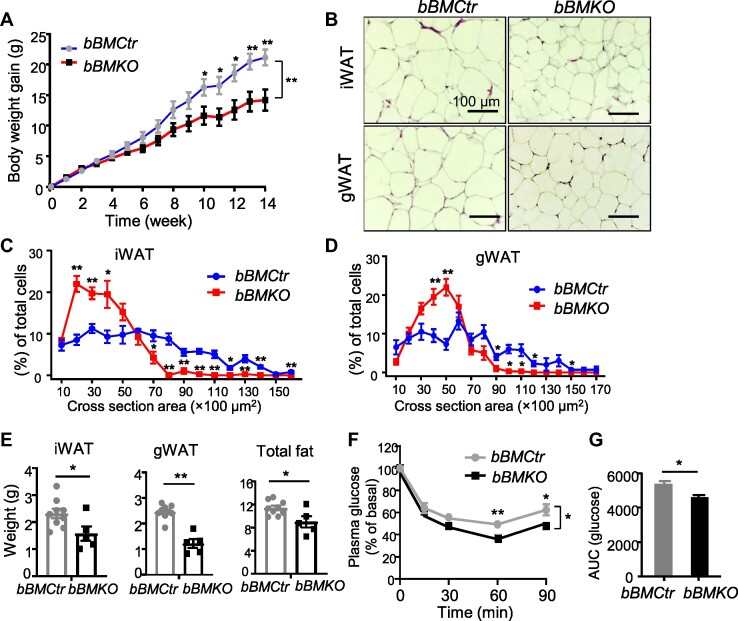
*Bmal1* deficiency in beige fat protects against HFD-induced obesity. (**A**) Body weight growth curve as shown by weekly weight gain of male *bBMCtr* (*n* = 9) and *bBMKO* (*n* = 5) mice fed with 45% HFD for 14 weeks. *P ≤ 0.05, **P ≤ 0.01, one-way ANOVA with post-hoc pairwise *t*-test. (**B**–**D**) H&E histology of iWAT and gWAT after HFD (**B**), with quantification of adipocyte size distribution (**C** and **D**). (**E**) Tissue weight analysis of iWAT, gWAT, and total fat mass in HFD-fed *bBMCtr* (*n* = 9) and *bBMKO* mice (*n* = 5). (**F** and **G**) Plasma glucose levels during ITT (**F**) with AUC quantification (**G**) in male *bBMCtr* (*n* = 6) and *bBMKO* mice (*n* = 5) after HFD feeding. *P ≤ 0.05, **P ≤ 0.01, by Student's *t*-test or ANOVA with post-hoc pairwise *t*-test.

To determine the effect of *bBMKO* beigeing on glucose homeostasis, we performed glucose tolerance test (GTT) and found that blood glucose levels at 60 and 120 min following an intraperitoneal bolus in *bBMKO* mice were significantly lower than that of the controls (Figure 4G), with area under the curve (AUC) reduced (Figure 4H). *bBMKO* mice also displayed augmented insulin-induced glucose-lowering effect at 90 min after stimulation (Figure 4I). Using hyperinsulinemic–euglycemic insulin clamp to achieve steady-state condition, we further examined glucose disposal in these mice. Both glucose infusion rate (GIR) and glucose disposal rate (GDR) were significantly elevated in *bBMKO* mice as compared to *bBMCtr* (Figure 4J). Enhanced glucose disposal was likely due to increased uptake in iWAT in *Bmal1*-deficient mice (Figure 4K), while the uptake in gWAT, BAT, or soleus muscle was comparable to controls (Figure 4K and L). Hepatic glucose production (HGP) at basal or clamped state were not significantly altered (Figure 4M). Direct examination of insulin signaling revealed augmented Akt phosphorylation in *Bmal1*-deficient iWAT upon insulin stimulation (Figure 4N). In contrast, Akt activation levels in gWAT of *bBMKO* and *bBMCtr* mice were similar (Figure 4O). Due to improved energy balance, we found that, on a high-fat diet (HFD), *bBMKO* mice maintained lower body weight than littermate controls (Figure 5A). iWAT and gWAT from HFD-fed *bBMKO* mice displayed reduced adipocyte hypertrophy as compared to *bBMCtr* (Figure 5B), with a marked shift toward smaller adipocytes as revealed by size distribution (Figure 5C and D). iWAT and gWAT fat pad weight and combined mass were reduced in *bBMKO* mice (Figure 5E). Moreover, *bBMKO* mice displayed augmented insulin sensitivity on HFD, as indicated by stronger glucose lowering stimulated by insulin than controls (Figure 5F and G).

### Beige fat Bmal1 deficiency dampens MRTF/SRF signaling with enhanced beige preadipocyte differentiation

**Figure 6:**
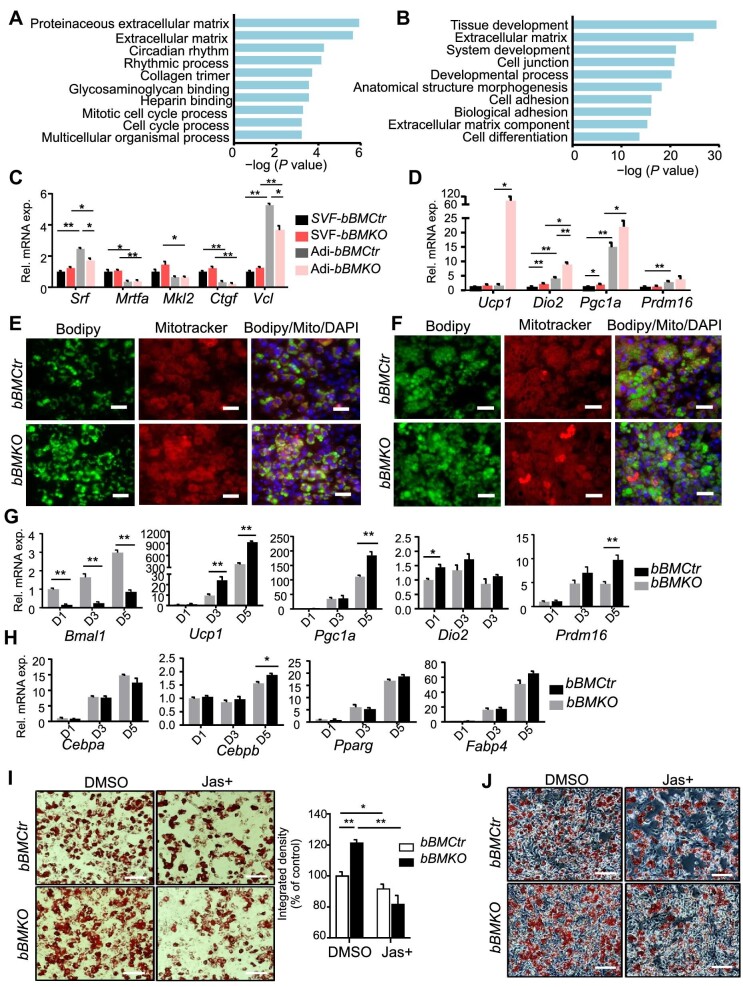
Loss of *Bmal1* suppresses MRTF/SRF pathway and promotes beige adipogenesis. (**A** and **B**) KEGG analysis of down-regulated pathways in iWAT of 6-week-old *bBMKO* mice as compared to *bBMCtr* mice at ZT3 (**A**, *n* = 3/genotype) and the overlap with processes suppressed by cold acclimation in *bBMCtr* mice (**B**, *n* = 3/genotype). (**C** and **D**) RT-qPCR analysis of genes in MRTF/SRF pathway (**C**) and the thermogenic program (**D**) in isolated SVF or mature adipocytes from iWAT of 6-week-old *bBMKO* and *bBMCtr* mice at ZT3 (*n* = 5–6/group). Adi, adipocytes. *P ≤ 0.05, **P ≤ 0.01, by Student's *t*-test. (**E** and **F**) Representative images of Bodipy (green) and Mitotracker (red) staining at Day 4 (**E**) and Day 6 (**F**) of beige adipogenic differentiation using SVF isolated from iWAT of 6-week-old *bBMCtr* (*n* = 5) and *bBMKO* (*n* = 5) mice at ZT3. Scale bar, 50 μm. (**G** and **H**) RT-qPCR analysis of thermogenic gene program (**G**) and adipogenic markers (**H**) at Days 1, 3, and 5 of beige adipogenic differentiation time course (*n* = 3/group). (**I** and **J**) Representative images of Oil-Red-O staining with quantification of staining (**I**) and phase-contrast images (**J**) of Day 5-differentiated beige adipocytes from iWAT SVF of 6-week-old *bBMKO* and *bBMCtr* mice at ZT3, with or without Jas (0.1 μM). Scale bar, 100 μm.

In *Bmal1*-deficient iWAT, extracellular matrix (ECM) and cell adhesion-related pathways were identified among the top down-regulated processes (Figure 6A), and these overlapped with cold acclimation-suppressed pathways (Figure 6B). *Bmal1* deficiency and cold-induced suppressions of these ECM-related processes were synergistic (Supplementary Figure S4A). Ingenuity pathway analysis identified SRF and MRTF-A as upstream regulators (Supplementary Figure S4B), with down-regulation of MRTF/SRF-positive transcriptional targets (Supplementary Figure S4C). *Srf* and *Mkl1* (*Mrtf-a*) expression levels were moderately reduced in *Bmal1*-dificient iWAT at ambient temperature and markedly suppressed by cold, while cold stimulation did not affect *Srf* or *Mkl1* in *bBMCtr* (Supplementary Figure S4D). As Prx1-Cre targets beige progenitors and mature adipocytes, we isolated stromal vascular fraction (SVF) containing preadipocytes and mature adipocytes from iWAT beige depot to determine Bmal1 modulation of cytoskeleton–MRTF/SRF cascade. Similar to Bmal1 silencing in mesenchymal progenitors, F-actin staining of primary preadipocytes isolated from *bBMKO* iWAT was markedly attenuated together with reduced cell size (Supplementary Figure S4E). Significant down-regulations of *Srf* and *Vcl* were found only in mature adipocytes in *Bmal1*-deficient iWAT (Figure 6C) but not in SVFs. Interestingly, *Srf* was induced in adipocytes as compared to SVF in *bBMCtr*, although *Mkl1* and *Mkl2* were suppressed together with reduced *Ctgf* expression suggesting attenuated MRTF/SRF transcription activity. In line with findings from iWAT, thermogenic program in *Bmal1*-deficient beige adipocytes, including *Ucp1, Dio2*, and *Pgc1*α, was induced (Figure 6D), whereas adipogenic gene expression was comparable to that of controls (Supplementary Figure S4F).

*bBMKO* iWAT displayed marked up-regulation of fat cell differentiation pathway (Supplementary Figure S4G). We postulated that impaired MRTF/SRF regulation due to loss of *Bmal1* may underlie the beigeing effect by promoting beige precursor differentiation. Upon beige induction, *Bmal1*-deficient SVF displayed abundant lipid accumulation with enhanced mitochondrial staining compared to *bBMCtr* cells (Figure 6E and F). Thermogenic genes, including *Ucp1, Pgc1*α, and *Dio2*, were induced in *Bmal1*-deficient SVFs (Figure 6G), together with elevated early brown lineage marker *Prdm16*. Genes involved in mitochondrial fatty acid transport and oxidation were up-regulated (Supplementary Figure S4H), while adipogenic gene expression were largely comparable to that of controls (Figure 6H) in line with findings from isolated adipocytes. To test attenuated MRTF/SRF pathway underlying *bBMKO* beige progenitor differentiation, we treated beige progenitors with Jasplakinolide (Jas), an actin polymerizing agent that activates MRTF/SRF transcription activity (Allingham et al., 2006). Consistent with its effect on MRTF/SRF activation, Jas inhibited beige adipogenic differentiation of SVF from *bBMCtr* iWAT (Figure 6I and J). Importantly, activating MRTF/SRF by Jas rescued the effect of loss of *Bmal1* on enhancing beige adipogenesis, as it was able to suppress differentiation of *Bmal1*-deficient SVF to a similar extent as observed in controls.

### Bmal1 overexpression in beige fat enhances MRTF/SRF signaling with impaired beigeing

**Figure 7:**
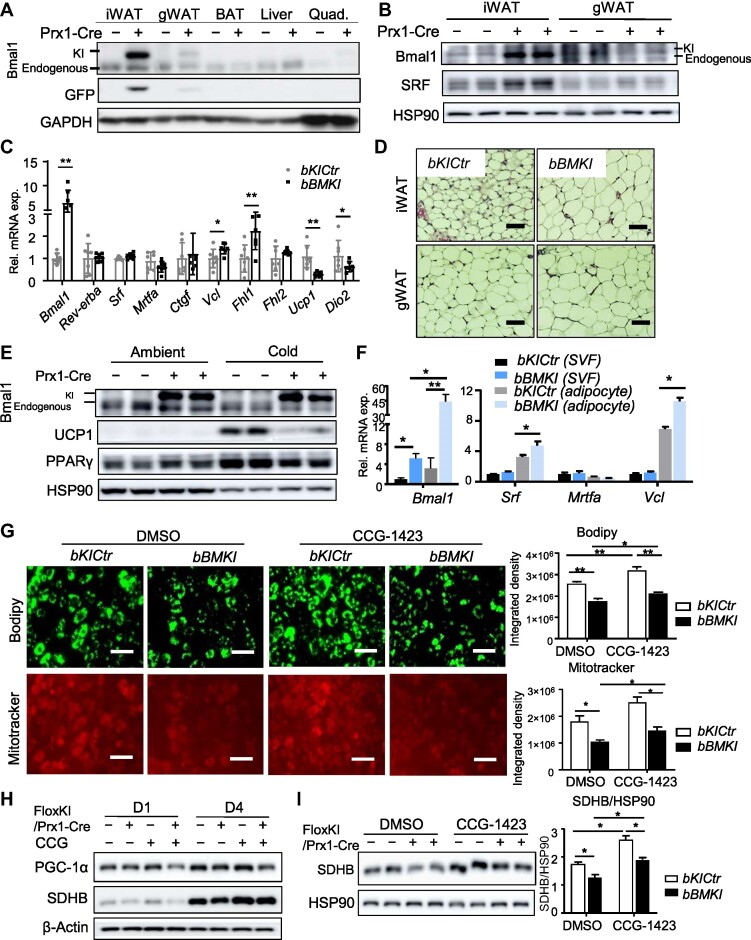
Beige fat-selective *Bmal1* overexpression promotes SRF pathway and suppresses thermogenic gene program. (**A**) Immunoblot analysis of knock-in (∼95 kDa) and endogenous Bmal1 (∼80 kDa) protein in female *bKICtr* and *bBMKI* mice. Each lane represents a pooled sample of *n* = 4–5 mice. (**B**) Immunoblot analysis of Bmal1 and SRF in iWAT and gWAT depots in female *bKICtr* and *bBMKI* mice. Each lane represents a pooled sample of *n* = 3 mice. (**C**) RT-qPCR analysis of MRTF/SRF pathway-related and thermogenic gene expression in iWAT of male *bKICtr* (*n* = 6) and *bBMKI* mice (*n* = 7). (**D**) H&E histology of iWAT and gWAT from 6-week-old male *bKICtr* and *bBMKI* mice. Scale bar, 50 μm. (**E**) Immunoblot analysis of thermogenic and adipogenic factors in *bKICtr* and *bBMKI* mice at ambient temperature or after cold acclimation. Each lane represents a pooled sample of *n* = 4–5 mice. (**F**) RT-qPCR analysis of MRTF/SRF pathway genes in isolated SVF or mature adipocytes from iWAT of *bKICtr* and *bBMKI* mice (*n* = 5–6/group). (**G**) Representative images of Bodipy (green) and Mitotracker staining (red) at Day 5 of beige differentiation of *bKICtr* and *bBMKI* SVF treated with CCG-1423 (5 μM) or DMSO, with quantification of Bodipy and Mitotracker staining. Scale bar, 70 μm. (**H** and **I**) Immunoblot analysis of *bKICtr* and *bBMKI* preadipocytes at Day 1 or Day 4 of beige differentiation treated with CCG-1423 (5 μM) or DMSO (**H**) and at Day 5 with quantification (**I**). Each lane represents a pooled sample of *n* = 3 replicates.

We generated a conditional *Bmal1* knock-in model via CRISPR-mediated gene targeting, consisting of a CMV promoter-driven Flag-tagged *Bmal1* with bi-cistronic GFP following a Lox–Stop–Lox cassette within ROSA26 locus (Supplementary Figure S5A), to determine whether the Bmal1–cytoskeleton–MRTF/SRF axis is sufficient to impact beige thermogenic capacity. Floxed knock-in (*bKICtr*) mice were crossed with Prx1-Cre for beige fat-selective *Bmal1* overexpression (*bBMKI*), with tagged Bmal1 protein (∼95 kDa) detected at ∼5–6 times higher than endogenous level (∼80 kDa) along with GFP (Figure 7A). Knock-in expression was barely detectable in gWAT and was not present in the BAT, liver, or skeletal muscle. Consistent with Bmal1 regulation of SRF, elevated SRF protein was found in iWAT but not gWAT in *bBMKI* mice (Figure 7B). SRF target genes, *Vcl* and *Fhl1*, were induced in *Bmal1*-overexpressing beige fat, while *Ucp1* and *Dio2* were suppressed (Figure 7C). iWAT in 6-week-old *bBMKI* mice lost beige characteristics observed in controls with marked adipocyte hypertrophy, and the histology resembled that of visceral fat (Figure 7D). Visceral fat in *bBMKI* mice displayed a tendency toward larger adipocytes. When subjected to cold, *bBMKI* iWAT lacked robust inductions of UCP1 and PPARγ found in age-matched *bKICtr* (Figure 7E). In isolated beige adipocytes from *bBMKI* mice, *Srf* and *Vcl* were significantly up-regulated, while their expression levels were comparable to controls within the SVF (Figure 7F). To test whether increased MRTF/SRF activity underlies impaired beigeing in *bBMKI* beige adipocytes, SVFs isolated from *bBMKI* were treated with CCG-1423, a specific inhibitor of MRTF/SRF-mediated transcription (Evelyn et al., 2007, 2010). *Bmal1* overexpression suppressed beige adipogenic differentiation of *bBMKI* SVF, consistent with enhanced MRTF/SRF signaling. CCG-1423 promoted beige differentiation of *bKICtr* cells, as indicated by Bodipy and Mitotracker staining and the quantitative analysis (Figure 7G). Notably, CCG-1423 induction of beige adipogenesis was abolished in Bmal1-overexpressing SVFs, suggesting that *Bmal1* gain-of-function confers resistance to MRTF/SRF inhibition. Furthermore, in agreement with Mitotracker staining, at Day 1 and Day 4 of beige adipogenic differentiation, PGC-1α and succinate dehydrogenase enzyme (SDHB) protein levels were induced by CCG-1423 in *bKICtr* cells indicative of increased mitochondrial abundance, but not sufficient in *bBMKI* cells (Figure 7H and I).

### Bmal1 overexpression in beige fat leads to adipocyte hypertrophy with impaired insulin sensitivity

**Figure 8:**
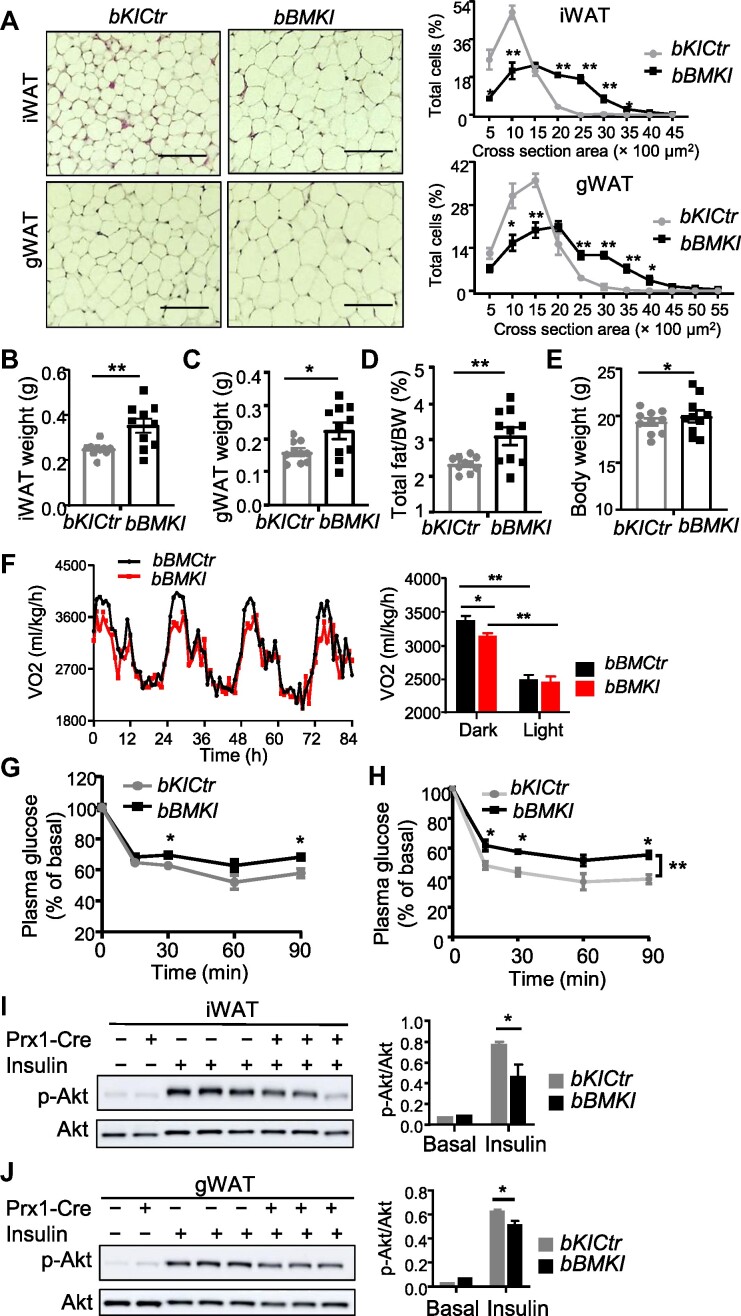
*Bmal1* overexpression in beige fat induces adipocyte hypertrophy and impairs insulin responsiveness. (**A**) H&E histology of iWAT and gWAT from 3-month-old male *bKICtr* and *bBMKI* mice with corresponding quantification of adipocyte size distribution. Scale bar, 100 μm. (**B**–**E**) Tissue weight analysis of iWAT (**B**), gWAT (**C**), total fat mass (**D**), and body weight (**E**) in 3-month-old *bKICtr* and *bBMK*I mice (*n* = 10/group). (**F**) Average tracing and quantitative analysis of oxygen consumption in 12-week-old *bKICtr* (*n* = 7) and *bBMKI* (*n* = 8) mice. *P ≤ 0.05, **P ≤ 0.01, by Student's *t*-test. (**G** and **H**) Plasma glucose levels during ITT (0.75 U/kg) in *bKICtr* and *bBMKI* mice under ambient temperature (**G**) or after cold acclimation at 10°C for 2 weeks (**H**). *n* = 6–7 mice/group. *P ≤ 0.05, **P ≤ 0.01, by one-way ANOVA with post-hoc pairwise *t*-test. (**I** and **J**) Immunoblot analysis of Akt phosphorylation in response to 0.5 U/kg insulin stimulation in iWAT (**I**) or gWAT (**J**) from female *bKICtr* and *bBMKI* mice with quantification. Each lane represents a pooled sample of *n* = 4–5 mice for basal and *n* = 3 mice for insulin-stimulated groups. *P ≤ 0.05 by student's *t*-test.

In 3-month-old *bBMKI* mice, adipocyte hypertrophy of iWAT and gWAT was evident (Figure 8A), with adipocyte size distribution shifted to larger adipocytes as compared to age-matched controls. iWAT and gWAT weights were increased ∼30% (Figure 8B and C), contributing to higher fat mass (Figure 8D) and body weight (Figure 8E). *bBMKI* BAT also displayed elevated lipid accumulation (Supplementary Figure S5B) with increased tissue weight (Supplementary Figure S5C). In addition, oxygen consumption in *bBMKI* mice was significantly elevated at the dark active phase (Figure 8F), together with higher activity level (Supplementary Figure S5D) without altering food intake (Supplementary Figure S5E). The loss of beigeing with resultant increased adiposity impaired insulin responsiveness in *bBMKI* mice, as shown by attenuated glucose-lowering response to insulin (Figure 8G), and the impairment of insulin response was more pronounced following cold acclimation (Figure 8H). Furthermore, in both subcutaneous beige and visceral fat depots, insulin stimulation of Akt phosphorylation was reduced in *bBMKI* as compared to *bKICtr* (Figure 8I and J). Taken together, *Bmal1* gain-of-function in beige fat depot is sufficient to suppress beigeing that negatively impacts whole-body metabolic homeostasis.

## Discussion

Actin cytoskeleton and MRTF/SRF activity are fundamental mechanisms in developmental processes (Connelly et al., 2010; Olson and Nordheim, 2010), while the inducible metabolic capacity of beige fat is a potential target to promote energy expenditure (Boss and Farmer, 2012). Our investigation defined a novel temporal control of actin dynamic and MRTF/SRF activity as an inhibitory pathway of beige precursor differentiation. Due to overlapping function and developmental origins, determining the contribution of adipose depot-intrinsic clocks to whole-body metabolism remains challenging. Using beige depot-selective gain- and loss-of-function models, our current study further delineated the contribution of circadian clock modulation of beige fat metabolic capacity to whole-body metabolic homeostasis.

Several findings in our study uncovered the circadian regulation of the actin cytoskeleton–MRTF/SRF signaling axis. Transcriptomic profiling of mesenchymal beige precursors uncovered Bmal1 transcriptional control of actin cytoskeleton and ECM-related processes, with *Bmal1* loss- or gain-of-function altering F-actin organization and SRF activity. Examination of Bmal1 chromatin association reveled its direct transcriptional control of genes within cytoskeleton–MRTF/SRF cascade. In beige fat depot, SRF and MRTF-A displayed diurnal oscillations, and MRTF-A/SRF transcriptional targets, particularly ECM–cytoskeleton-related pathways, were down-regulated in *Bmal1*-deficient iWAT. We focused on Bmal1 functional targets within the actin cytoskeleton–MRTF/SRF pathway based on these findings. It is conceivable that there are additional clock targets within this signaling network, and other clock regulators within the clock circuit may function in concert with Bmal1 to drive the circadian oscillations in actin dynamic and SRF/MRTF-A activity.

F-actin polymerization and the resultant MRTF nuclear translocation for transcriptional activation of SRF transduce extracellular biochemical or physical niche signals, including growth factors, cytokines, and cell–matrix or cell–cell interactions (Miralles et al., 2003; Esnault et al., 2014). MRTF/SRF transcriptional response, via canonical CArG response elements (Selvaraj and Prywes, 2004; Sun et al., 2006), drives various cellular behaviors invovled in stem cell lineage determination and differntiaton in tissue growth and development processes (Olson and Nordheim, 2010). The circadian control that we identified raises the possibility of time-of-the-day variations of signaling events within the actin–MRTF/SRF cascade or cell–matrix interactions upstream of actin remodeling (Olson and Nordheim, 2010; Xiong et al., 2022), implicating a potential role of circadian clock in orchestrating cytoskeleton–MRTF/SRF signaling transduction during beige adipocyte development. Serum induces clock synchronization in the liver involving actin remodeling and MRTF/SRF activation (Gerber et al., 2013), while transcriptional regulation of actin cytoskeleton dynamics by the clock circuit is not known. Given prior observation of MRTF/SRF-induced clock entrainment (Gerber et al., 2013; Esnault et al., 2014; Xiong et al., 2022), our finding of Bmal1 transcriptional control in actin–MRTF/SRF cascade suggests a regulatory feedback loop between the circadian clock and cytoskeleton-related processes. How a potential feedback loop between the circadian clock and cytoskeleton–MRTF/SRF may modulate adipocyte biology remains to be explored.

As intracellular actin dynamic and the MRTF/SRF transcriptional response transduce extracellular niche signals (Olson and Nordheim, 2010), a reciprocal interactions between the circadian clock and actin–MRTF/SRF axis may facilitate cellular entrainment and adaptation to cyclic cues within its extracellular microenvironment (Xiong et al., 2022). Little is known to date regarding interactions between circadian clock, extracellular niche, and beige adipocyte development. *Bmal1* deficiency in beige adipocytes, via its regulation of actin cytoskeleton–MRTF/SRF activity, induced the thermogenic program, whereas its overexpression suppressed beigeing with adipocyte hypertrophy. These findings, in aggregate, suggest a possibility that clock modulation of beigeing as we observed may involve the actin–MRTF/SRF cascade determining thermogenic induction and energy storage via adipogenesis in beige precursor cells (McDonald et al., 2015; Lin et al., 2018). Circadian clock exerts temporal coordination of metabolic processes, and its disruption predisposes to the risk for obesity and insulin resistance (Panda et al., 2002; Turek et al., 2005). While MRTF/SRF suppresses beige adipogenesis, recent studies further demonstrated that MRTF/SRF activity was induced in HFD-induced obesity, promoting an adipogenic progenitor fate switch to fibrogenic lineage that predisposes to adipose fibrosis (McDonald et al., 2015; Lin et al., 2018). We found in previous studies that mice subjected to shiftwork developed marked adipocyte hypertrophy with extensive fibrosis in both subcutaneous and visceral fat depots (Xiong et al., 2021). Thus, a clock–actin–MRTF/SRF regulatory pathway may also function in classical WAT to modulate fibrotic changes that could be applicable to obesity or clock disruption-related metabolic diseases. With the wide-spread circadian misalignment of a modern lifestyle, our findings may spur future investigations of potential circadian etiologies involving the clock–MRTF/SRF axis in clock disruption-associated metabolic consequences.

In conclusion, our study identified the clock–actin–MRTF/SRF regulatory axis as an inhibitory mechanism of beige adipocyte development, and clock modulation of beige fat thermogenic capacity contributes to energy homeostasis (Logan et al., 2002; Krueger et al., 2014; Sanchez-Gurmaches et al., 2015). This mechanistic link between clock regulation and beige thermogenic function adds to our current understanding of temporal control in metabolic regulations.

## Materials and methods

### Animals

Mice were maintained in the City of Hope vivarium under a constant 12:12 light/dark cycle, with lights on at 6:00 AM (ZT0). All animal experiments were approved by the Institutional Animal Care & Use Committee (IACUC) of City of Hope. Experimental procedures were carried out in accordance with IACUC approval. *Bmal1*^fl/fl^ and Prx1-Cre transgenic mice were obtained from the Jackson Laboratory (Logan et al., 2002; Storch et al., 2007). Conditional ROSA-26 *Bmal1* knock-in mice were generated via CRISPR-mediated gene targeting by Cyagen Biosciences. The targeted allele is shown in Supplementary Figure S5A. Genotyping primers are listed in Supplementary Table S4. Ambient temperature was set at 21°C. Cold acclimation was performed in 10- to 12-week-old mice at 10°C for 2 weeks using environmental chamber with programmable temperature control (Power Scientifics). Thermoneutral adaptation was carried out for 2 weeks at 30°C. Both male and female mice were used as indicated in the experiments.

### C3H10T1/2 culture, generation of stable cell lines, and beige differentiation

C3H10T1/2 cells were obtained from ATCC, maintained, and differentiated in Dulbecco's modified Eagle medium (DMEM) with 10% fetal bovine serum (FBS), as previously described (Nam et al., 2015b; Liu et al., 2020). Stable lines of *Bmal1* knockdown or overexpression were generated as previously described (Nam et al., 2015b). For beige adipogenic differentiation, induction medium (1.6 μM insulin, 1 μM dexamethasone, 0.5 mM IBMX, 0.5 μM Rosiglitazone, and 1 nM T3) was used for 3 days followed by maintenance medium for 6 days with insulin, Rosi, and T3 (Liu et al., 2020).

### Primary preadipocyte isolation and beige differentiation

The SVFs containing preadipocytes were isolated from subcutaneous or gonadal fat pads, as described (Chatterjee et al., 2013). Briefly, dissected fat pads were cut into small pieces and digested in 0.1% collagenase type 1 in DMEM with 0.8% bovine serum albumin at 37°C in a horizontal shaker for 60 min. The digestion mixture was passed through Nylon mesh and centrifuged to collect the top adipocyte layer and the pellet containing the SVF with preadipocytes. Preadipocytes were cultured in F12/DMEM supplemented with bFGF (2.5 ng/ml), expanded for two passages, and subjected to differentiation in collagen type I-coated 6-well plates at 90% confluency. Beige adipocyte differentiation was induced for 2 days in induction medium (10% FBS, 1.6 μM insulin, 1 μM dexamethasone, 0.5 mM IBMX, 0.5 μM Rosi, and 1 nM T3) before switching to maintenance medium for 4 days (insulin, Rosi, and T3). Jas (0.1 μM) and CCG-1423 (5 μM) were obtained from Cayman Chemicals and added for the entire differentiation time course.

### Oil-red-O, Bodipy, and Mitotracker staining

These staining procedures were performed as previously described (Nam et al., 2015b). Briefly, for Oil-red-O staining, cells were fixed using 10% formalin and 0.5% Oil-red-O solution was incubated for 1 h. Bodipy 493/503 was used at 1 mg/L together with DAPI for 15 min, following 4% paraformaldehyde fixation and permeabilization with 0.2% Triton X-100. Mitotracker Deep Red FM was applied at 100 nM to differentiated adipocytes and incubated for 30 min prior to fixation (Nam et al., 2015a; Liu et al., 2020).

### Immunoblot analysis

Total protein (20–40 μg) was resolved on sodium dodecyl sulfate–polyacrylamide gel electrophoresis followed by immunoblotting after nitrocellulose membrane transfer. Membranes were developed by chemiluminescence (Supersignal; Pierce Biotechnology). Antibodies used are listed in Supplementary Table S1.

### RNA extraction and RT-qPCR analysis

Trizol (Invitrogen) or RNeasy miniprep kits (Qiagen) were used to isolate total RNA from snap-frozen tissues or cells, respectively. cDNA was generated using q-Script cDNA Supermix kit (Quanta Biosciences) and qPCR was performed in triplicate on 7500 Fast Real-Time PCR system (Applied Biosystems) with Perfecta SYBR Green Supermix (Quanta Biosciences). Relative expression levels were determined using the comparative Ct method with normalization to 36B4 as internal control. PCR primer sequences are listed in Supplementary Table S2.

### RNA-seq and computational analysis

RNA-seq reads were aligned with STAR software to the mouse reference genome mm10, and unique reads were quantified using HTSeq-count with GENCODE annotations (Anders et al., 2015). The RNA-seq reads were normalized and log-transformed using limma edgeR packages. *P*-values and the false discovery rate (FDR) were calculated from raw counts using DESeq2 (Love et al., 2014). Fold-change > 1.5, 50% fragments per kilobase of transcript per million mapped reads (FPKM) >0.1, unadjusted *P*-value <0.05, and FDR < 0.25 were used as cut-off for differentially expressed genes. Global analysis heatmaps were produced using heatmap.3 and the gplots package in R, using log2(FPKM + 0.1) values. The Pearson dissimilarity (1–Pearson correlation coefficient) was used as the distance metric for hierarchical clustering of rows and columns. Expression was centered so that each gene had a mean of 0, and centered expression was capped at –3 and 3, for clustering and visualization. RNA-seq dataset was deposited in NCBI GEO GSE183000.

### Hematoxylin–eosin histology and adipocyte size distribution

Adipose tissues were fixed with 10% neutral-buffered formalin for 72 h prior to embedding. Then, 10-μm paraffin sections were cut and processed for hematoxylin–eosin (H&E) staining. Adipocyte size area was measured by outlining the adipocytes, and the average of five representative 10× fields from each mouse was plotted for cross-sectional area distribution as described in Xiong et al. (2021).

### GTT, ITT, and insulin signaling examination

GTT and insulin tolerance test (ITT) were performed as described (Yin et al., 2020). Mice were fasted overnight prior to GTT with indicated doses of glucose administered via intraperitoneal injection. ITT was performed following 4 h of fasting with doses specified. Insulin signaling in adipose tissues was determined at baseline in mice fasted for 4 h, and insulin-stimulated tissues were collected 20 min following intraperitoneal 0.5 U/kg insulin injection.

### Hyperinsuinemic–euglycemic clamp study

The low-dose hyperinsuinemic–euglycemic clamp studies were performed as described (Yin et al., 2020). Mice were cannulated through the right jugular vein, allowed 4 days of recovery, and studied in a conscious state. Briefly, a priming dose (10 μCi) and a constant intravenous dose (0.1 μCi/min) of ^3^H-glucose were infused through the venous canula. Basal glucose production (BGP) was assessed after 50 min of glucose infusion. Mice were then primed with a bolus insulin followed by continuous infusion at 3 mU/kg/min. During this time, glucose was simultaneously infused to maintain a steady level at 100–140 mg/dl. For the analysis of insulin-stimulated glucose tissue uptake, 2-deoxy-D-[1-^14^C] glucose was administered as a bolus 45 min prior to completion of the clamp study. Individual tissues were snap-frozen and ^14^C-glucose uptake was assayed by scintillation counting and calculated from ^14^C-glucose plasma profile fitted with a double exponential curve and tissue content.

### Indirect calorimetry by Comprehensive Laboratory Animal Monitoring

Mice were single housed and acclimated in metabolic cages using the Comprehensive Laboratory Animal Monitoring System (Columbus Instruments), with ad libitum food and water with controlled lighting for 2 days prior to metabolic recording. Metabolic parameters, including oxygen consumption, CO_2_ production, respiratory exchange ratio, ambulatory activity, and food intake were recorded for 5 consecutive days as described in Yin et al. (2020).

### ChIP–qPCR and serum shock

Following formaldehyde fixation, C3H10T1/2 chromatin was sonicated and used for immunoprecipitation with Bmal1 antibody (Abcam AB93806) or IgG using Magna ChIP A/G kit (Millipore), as previously described (Chatterjee et al., 2013). Real-time PCR was carried out in triplicate using primers for Bmal1-binding E- or E’-box elements identified within ±2 kb regulatory regions using TRANSFAC. Primers for *Nr1d1* promoter E-box were used as a positive control, and upstream primers as negative control. Values were expressed as fold enrichment over IgG control normalized to 1% of input. ChIP–qPCR primers are listed in Supplementary Table S3. Serum shock synchronization in C3H10T1/2 was induced by 20% FBS treatment for 2 h following serum starvation overnight as previously described (Chatterjee et al., 2013).

### Statistical analysis

Data are expressed as mean ± standard error of the mean. Statistical analysis was performed using GraphPad Prism. The differences between groups were determined by unpaired two-tailed Student's *t*-test or one-way analysis of variance (ANOVA) with post-hoc pairwise *t*-tests with Bonferroni correction. A minimum of three biological replicates were used to perform statistical analysis. *P*-values ≤ 0.05 were considered statistically significant.

## Supplementary Material

mjac079_Supplemental_File

## Notes

**Author contributions:** X.X., W.L., and R.L.: data curation, formal analysis, methodology, and investigation; V.Y.: data curation, manuscript review and editing, and funding acquisition; K.M.: data curation, formal analysis, project administration, manuscript writing and editing, and funding acquisition.
