# Supplementary material for: Circadian clock control of MRTF/SRF pathway suppresses beige adipocyte thermogenic recruitment
Source: J Mol Cell Biol. 2022 Dec 29;14(12):mjac079. doi: 10.1093/jmcb/mjac079 (PMC10174720; doi:10.1093/jmcb/mjac079)
Supplement: mjac079_Supplemental_File [file mjac079_supplemental_file.pptx]

## Slide 1
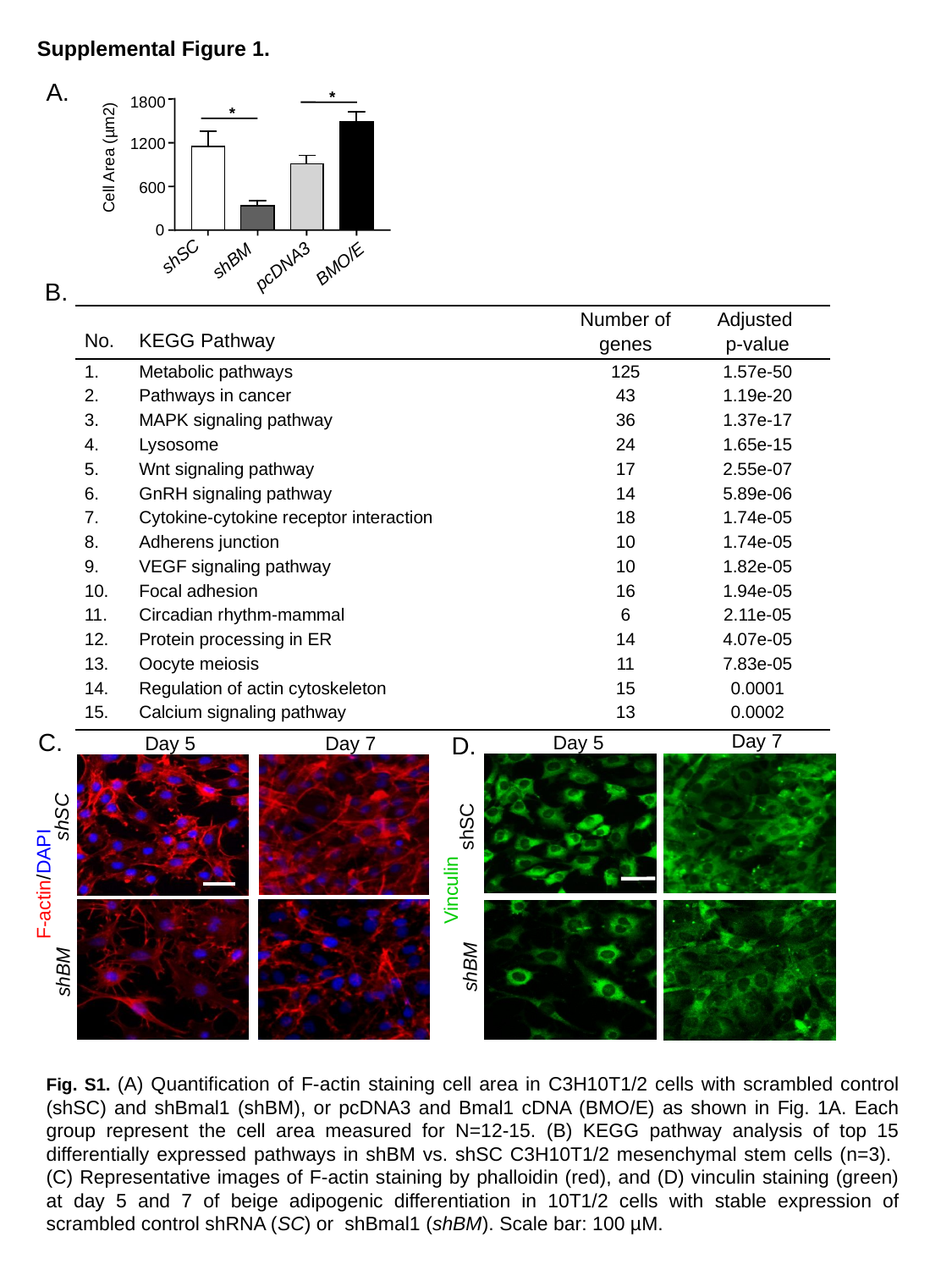

Supplemental Figure 1.
A.
*
*
shSC
shBM
BMO/E
pcDNA3
1800
1200
600
0
Cell Area (µm2)
B.
| No. | KEGG Pathway | Number of genes | Adjusted p-value |
| --- | --- | --- | --- |
| 1. | Metabolic pathways | 125 | 1.57e-50 |
| 2. | Pathways in cancer | 43 | 1.19e-20 |
| 3. | MAPK signaling pathway | 36 | 1.37e-17 |
| 4. | Lysosome | 24 | 1.65e-15 |
| 5. | Wnt signaling pathway | 17 | 2.55e-07 |
| 6. | GnRH signaling pathway | 14 | 5.89e-06 |
| 7. | Cytokine-cytokine receptor interaction | 18 | 1.74e-05 |
| 8. | Adherens junction | 10 | 1.74e-05 |
| 9. | VEGF signaling pathway | 10 | 1.82e-05 |
| 10. | Focal adhesion | 16 | 1.94e-05 |
| 11. | Circadian rhythm-mammal | 6 | 2.11e-05 |
| 12. | Protein processing in ER | 14 | 4.07e-05 |
| 13. | Oocyte meiosis | 11 | 7.83e-05 |
| 14. | Regulation of actin cytoskeleton | 15 | 0.0001 |
| 15. | Calcium signaling pathway | 13 | 0.0002 |
C.
Day 7
Day 5
shSC
Vinculin
shBM
D.
Day 5
Day 7
shSC
F-actin/DAPI
shBM
Fig. S1. (A) Quantification of F-actin staining cell area in C3H10T1/2 cells with scrambled control (shSC) and shBmal1 (shBM), or pcDNA3 and Bmal1 cDNA (BMO/E) as shown in Fig. 1A. Each group represent the cell area measured for N=12-15. (B) KEGG pathway analysis of top 15 differentially expressed pathways in shBM vs. shSC C3H10T1/2 mesenchymal stem cells (n=3). (C) Representative images of F-actin staining by phalloidin (red), and (D) vinculin staining (green) at day 5 and 7 of beige adipogenic differentiation in 10T1/2 cells with stable expression of scrambled control shRNA (SC) or shBmal1 (shBM). Scale bar: 100 µM.

## Slide 2
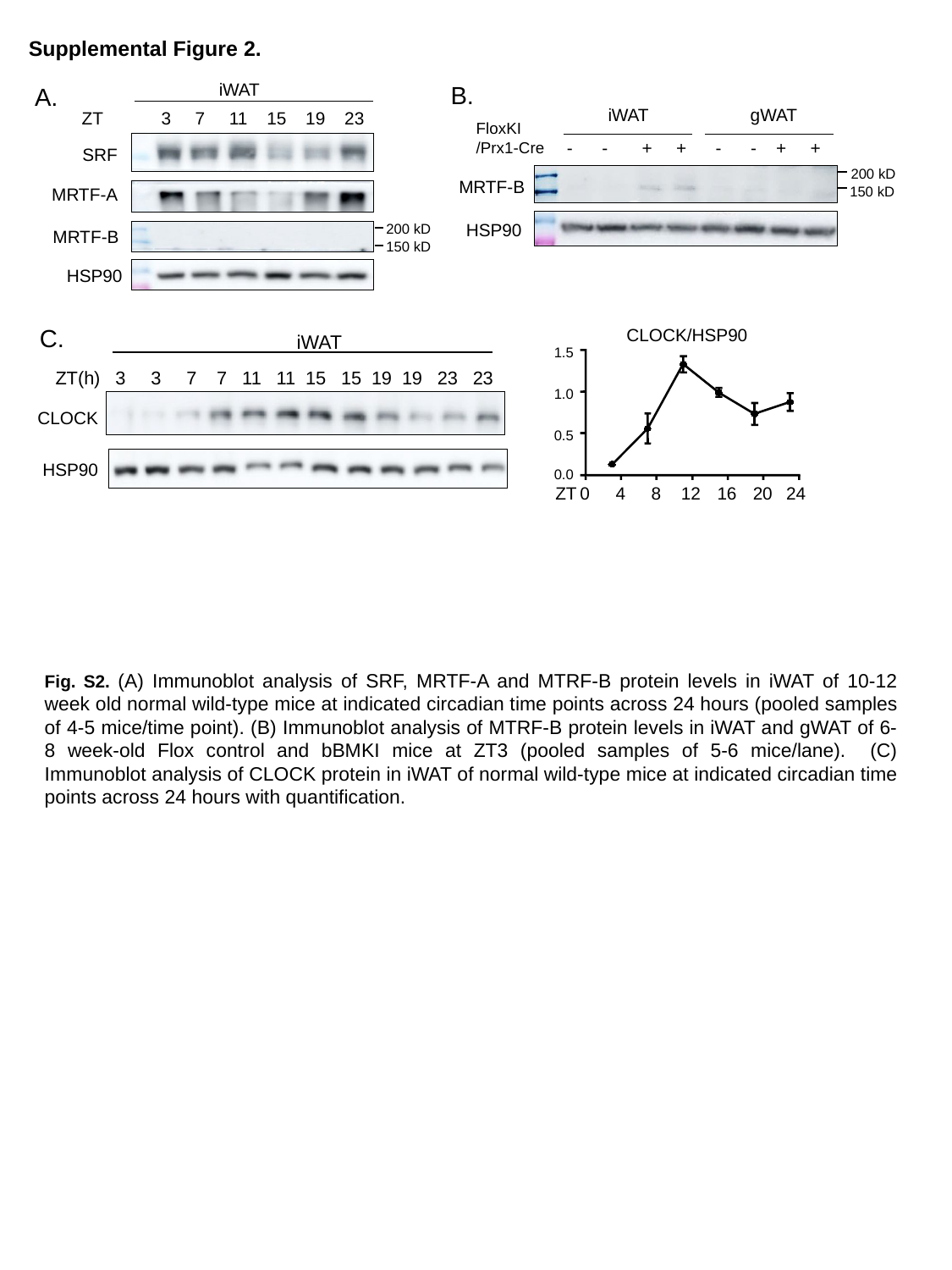

Supplemental Figure 2.
iWAT
B.
A.
 iWAT gWAT
- - + + - - + +
MRTF-B
HSP90
FloxKI
/Prx1-Cre
200 kD
150 kD
 ZT 3 7 11 15 19 23
SRF
MRTF-A
200 kD
MRTF-B
150 kD
HSP90
C.
iWAT
CLOCK/HSP90
1.5
1.0
0.5
0.0
24
0
4
8
12
16
20
ZT
ZT(h) 3 3 7 7 11 11 15 15 19 19 23 23
CLOCK
HSP90
Fig. S2. (A) Immunoblot analysis of SRF, MRTF-A and MTRF-B protein levels in iWAT of 10-12 week old normal wild-type mice at indicated circadian time points across 24 hours (pooled samples of 4-5 mice/time point). (B) Immunoblot analysis of MTRF-B protein levels in iWAT and gWAT of 6-8 week-old Flox control and bBMKI mice at ZT3 (pooled samples of 5-6 mice/lane). (C) Immunoblot analysis of CLOCK protein in iWAT of normal wild-type mice at indicated circadian time points across 24 hours with quantification.

## Slide 3
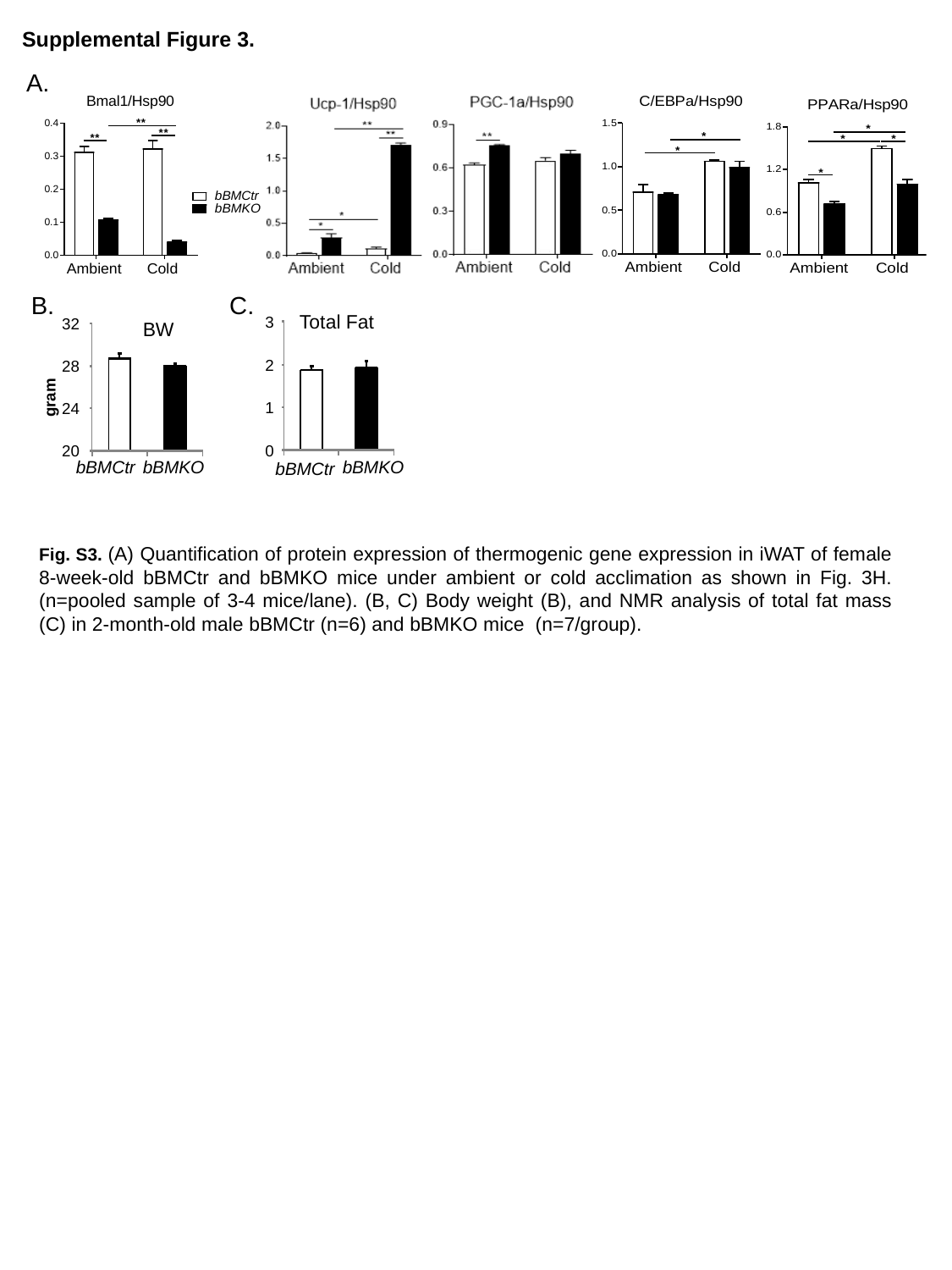

Supplemental Figure 3.
A.
B.
C.
Total Fat
3
2
1
0
bBMKO
bBMCtr
32
BW
28
gram
24
20
bBMCtr
bBMKO
Fig. S3. (A) Quantification of protein expression of thermogenic gene expression in iWAT of female 8-week-old bBMCtr and bBMKO mice under ambient or cold acclimation as shown in Fig. 3H. (n=pooled sample of 3-4 mice/lane). (B, C) Body weight (B), and NMR analysis of total fat mass (C) in 2-month-old male bBMCtr (n=6) and bBMKO mice (n=7/group).

## Slide 4
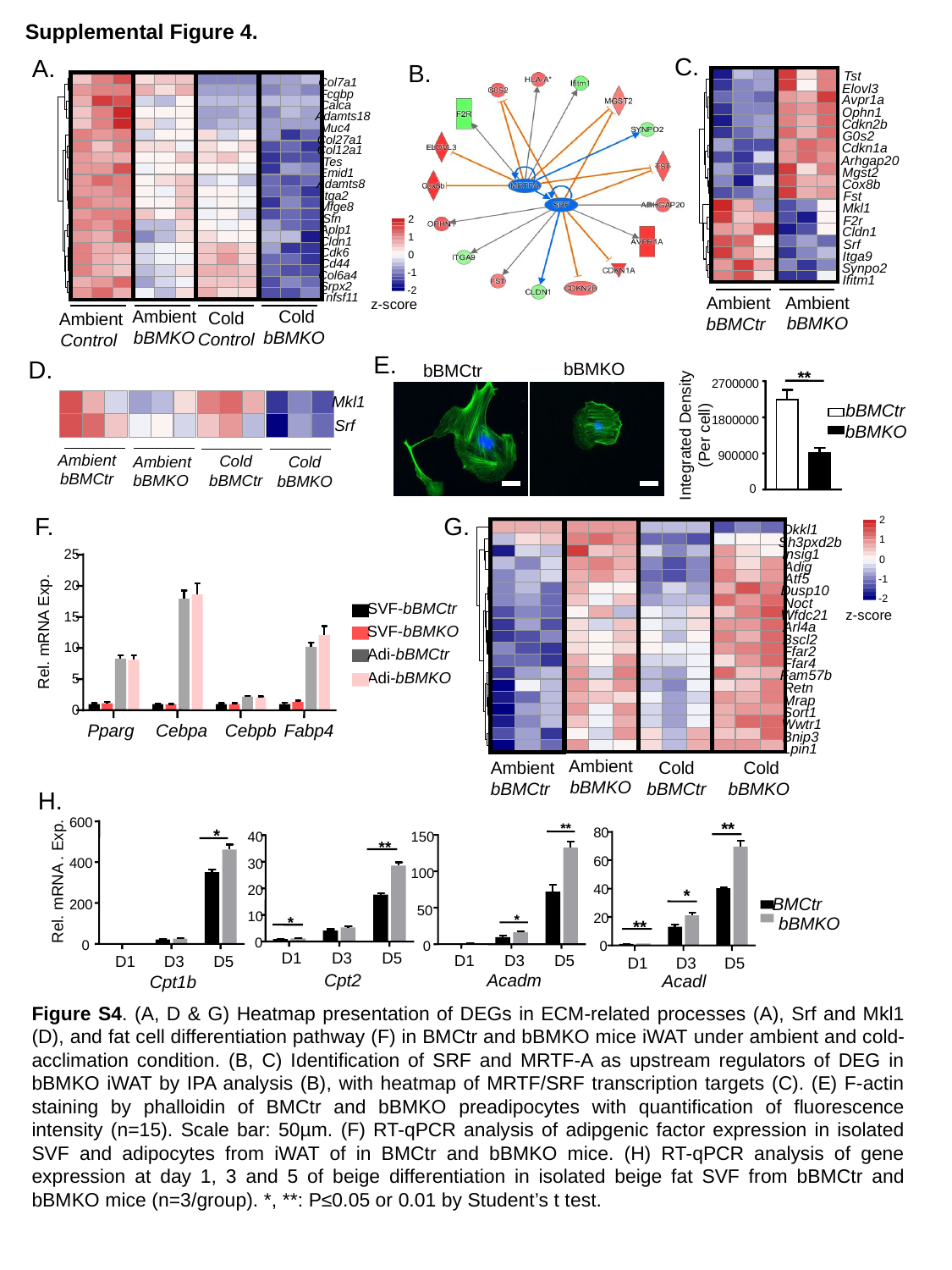

Supplemental Figure 4.
C.
A.
B.
Tst
Elovl3
Avpr1a
Ophn1
Cdkn2b
G0s2
Cdkn1a
Arhgap20
Mgst2
Cox8b
Fst
Mkl1
F2r
Cldn1
Srf
Itga9
Synpo2
Ifitm1
Ambient
bBMKO
Ambient
bBMCtr
Col7a1
Fcgbp
Calca
Adamts18
Muc4
Col27a1
Col12a1
Tes
Emid1
Adamts8
Itga2
Mfge8
Sfn
Aplp1
Cldn1
Cdk6
Cd44
Col6a4
Srpx2
Tnfsf11
Cold
bBMKO
Ambient
bBMKO
Cold
 Control
Ambient
Control
2
1
0
-1
-2
z-score
E.
D.
bBMKO
bBMCtr
**
2700000
1800000
900000
0
Integrated Density
(Per cell)
Mkl1
Srf
Ambient
bBMCtr
Ambient bBMKO
Cold bBMCtr
Cold bBMKO
bBMCtr
bBMKO
G.
F.
2
1
0
-1
-2
z-score
Dkkl1
Sh3pxd2b
Insig1
Adig
Atf5
Dusp10
Noct
Wfdc21
Arl4a
Bscl2
Ffar2
Ffar4
Fam57b
Retn
Mrap
Sort1
Wwtr1
Bnip3
Lpin1
Ambient
bBMKO
Cold
bBMKO
Cold
 bBMCtr
Ambient
bBMCtr
25
20
15
10
5
0
Pparg
Cebpa
Cebpb
Fabp4
SVF-bBMCtr
SVF-bBMKO
Adi-bBMCtr
Adi-bBMKO
Rel. mRNA Exp.
H.
600
*
400
Rel. mRNA . Exp.
200
0
D1
D3
D5
Cpt1b
**
80
60
40
*
20
**
0
D1
D3
D5
Acadl
**
150
100
50
*
0
D1
D3
D5
Acadm
40
**
30
20
10
*
0
D1
D3
D5
Cpt2
BMCtr
bBMKO
Figure S4. (A, D & G) Heatmap presentation of DEGs in ECM-related processes (A), Srf and Mkl1 (D), and fat cell differentiation pathway (F) in BMCtr and bBMKO mice iWAT under ambient and cold-acclimation condition. (B, C) Identification of SRF and MRTF-A as upstream regulators of DEG in bBMKO iWAT by IPA analysis (B), with heatmap of MRTF/SRF transcription targets (C). (E) F-actin staining by phalloidin of BMCtr and bBMKO preadipocytes with quantification of fluorescence intensity (n=15). Scale bar: 50µm. (F) RT-qPCR analysis of adipgenic factor expression in isolated SVF and adipocytes from iWAT of in BMCtr and bBMKO mice. (H) RT-qPCR analysis of gene expression at day 1, 3 and 5 of beige differentiation in isolated beige fat SVF from bBMCtr and bBMKO mice (n=3/group). *, **: P≤0.05 or 0.01 by Student’s t test.

## Slide 5
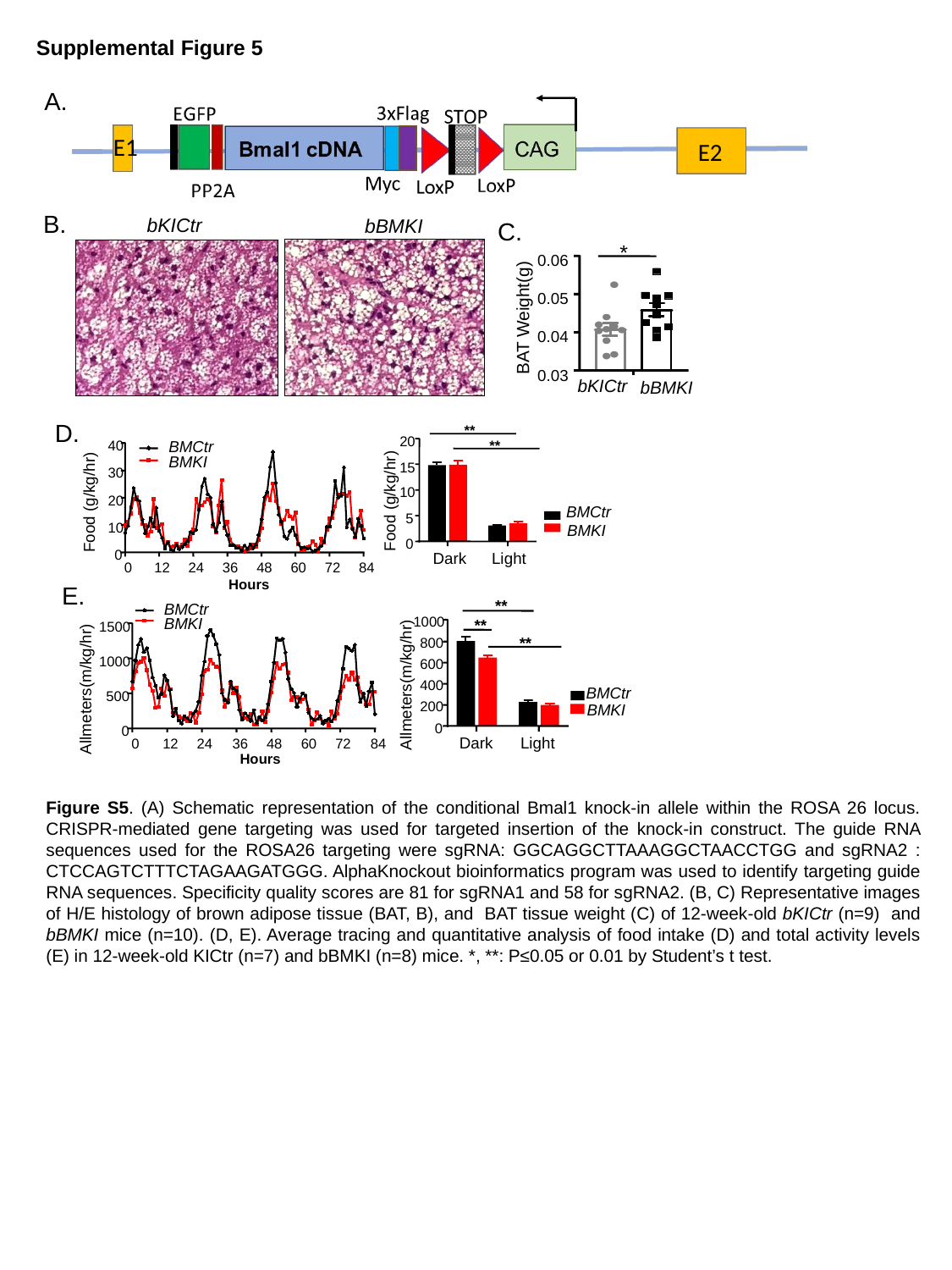

Supplemental Figure 5
A.
E1
E2
B.
C.
bKICtr
bBMKI
*
0.06
0.05
0.04
0.03
BAT Weight(g)
bKICtr
bBMKI
D.
**
**
Dark
Light
20
15
10
5
0
BMCtr
BMKI
40
30
20
10
0
0
12
24
36
48
60
72
84
Hours
Food (g/kg/hr)
Food (g/kg/hr)
BMCtr
BMKI
E.
**
1000
800
600
400
200
0
**
**
Dark
Light
BMCtr
BMKI
1500
1000
500
0
0
12
24
36
48
60
72
84
Hours
Allmeters(m/kg/hr)
Allmeters(m/kg/hr)
BMCtr
BMKI
Figure S5. (A) Schematic representation of the conditional Bmal1 knock-in allele within the ROSA 26 locus. CRISPR-mediated gene targeting was used for targeted insertion of the knock-in construct. The guide RNA sequences used for the ROSA26 targeting were sgRNA: GGCAGGCTTAAAGGCTAACCTGG and sgRNA2 : CTCCAGTCTTTCTAGAAGATGGG. AlphaKnockout bioinformatics program was used to identify targeting guide RNA sequences. Specificity quality scores are 81 for sgRNA1 and 58 for sgRNA2. (B, C) Representative images of H/E histology of brown adipose tissue (BAT, B), and BAT tissue weight (C) of 12-week-old bKICtr (n=9) and bBMKI mice (n=10). (D, E). Average tracing and quantitative analysis of food intake (D) and total activity levels (E) in 12-week-old KICtr (n=7) and bBMKI (n=8) mice. *, **: P≤0.05 or 0.01 by Student’s t test.

## Slide 6
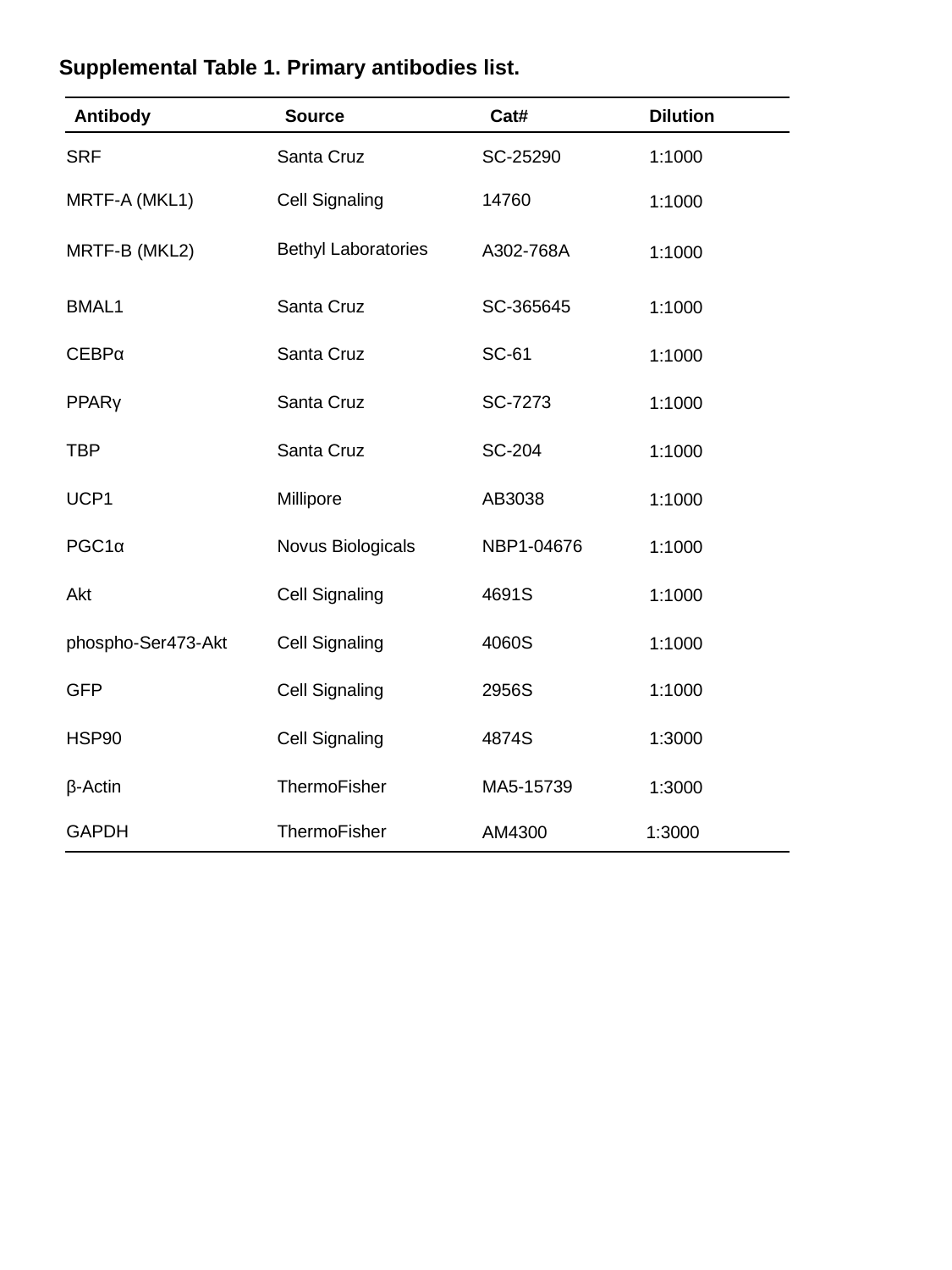

Supplemental Table 1. Primary antibodies list.
| Antibody | Source | Cat# | Dilution |
| --- | --- | --- | --- |
| SRF | Santa Cruz | SC-25290 | 1:1000 |
| MRTF-A (MKL1) | Cell Signaling | 14760 | 1:1000 |
| MRTF-B (MKL2) | Bethyl Laboratories | A302-768A | 1:1000 |
| BMAL1 | Santa Cruz | SC-365645 | 1:1000 |
| CEBPα | Santa Cruz | SC-61 | 1:1000 |
| PPARγ | Santa Cruz | SC-7273 | 1:1000 |
| TBP | Santa Cruz | SC-204 | 1:1000 |
| UCP1 | Millipore | AB3038 | 1:1000 |
| PGC1α | Novus Biologicals | NBP1-04676 | 1:1000 |
| Akt | Cell Signaling | 4691S | 1:1000 |
| phospho-Ser473-Akt | Cell Signaling | 4060S | 1:1000 |
| GFP | Cell Signaling | 2956S | 1:1000 |
| HSP90 | Cell Signaling | 4874S | 1:3000 |
| β-Actin | ThermoFisher | MA5-15739 | 1:3000 |
| GAPDH | ThermoFisher | AM4300 | 1:3000 |

## Slide 7
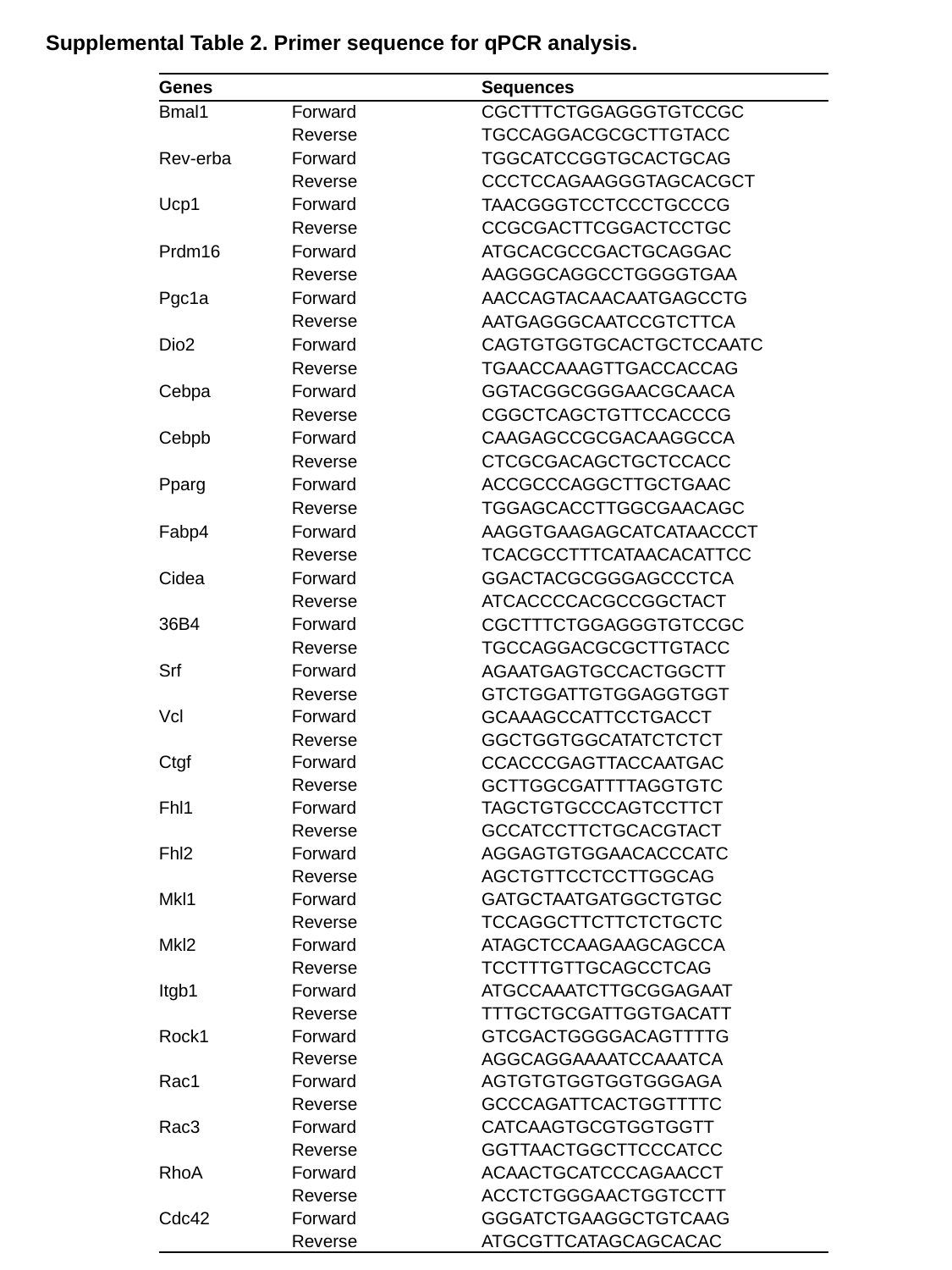

Supplemental Table 2. Primer sequence for qPCR analysis.
| Genes | | Sequences |
| --- | --- | --- |
| Bmal1 | Forward | CGCTTTCTGGAGGGTGTCCGC |
| | Reverse | TGCCAGGACGCGCTTGTACC |
| Rev-erba | Forward | TGGCATCCGGTGCACTGCAG |
| | Reverse | CCCTCCAGAAGGGTAGCACGCT |
| Ucp1 | Forward | TAACGGGTCCTCCCTGCCCG |
| | Reverse | CCGCGACTTCGGACTCCTGC |
| Prdm16 | Forward | ATGCACGCCGACTGCAGGAC |
| | Reverse | AAGGGCAGGCCTGGGGTGAA |
| Pgc1a | Forward | AACCAGTACAACAATGAGCCTG |
| | Reverse | AATGAGGGCAATCCGTCTTCA |
| Dio2 | Forward | CAGTGTGGTGCACTGCTCCAATC |
| | Reverse | TGAACCAAAGTTGACCACCAG |
| Cebpa | Forward | GGTACGGCGGGAACGCAACA |
| | Reverse | CGGCTCAGCTGTTCCACCCG |
| Cebpb | Forward | CAAGAGCCGCGACAAGGCCA |
| | Reverse | CTCGCGACAGCTGCTCCACC |
| Pparg | Forward | ACCGCCCAGGCTTGCTGAAC |
| | Reverse | TGGAGCACCTTGGCGAACAGC |
| Fabp4 | Forward | AAGGTGAAGAGCATCATAACCCT |
| | Reverse | TCACGCCTTTCATAACACATTCC |
| Cidea | Forward | GGACTACGCGGGAGCCCTCA |
| | Reverse | ATCACCCCACGCCGGCTACT |
| 36B4 | Forward | CGCTTTCTGGAGGGTGTCCGC |
| | Reverse | TGCCAGGACGCGCTTGTACC |
| Srf | Forward | AGAATGAGTGCCACTGGCTT |
| | Reverse | GTCTGGATTGTGGAGGTGGT |
| Vcl | Forward | GCAAAGCCATTCCTGACCT |
| | Reverse | GGCTGGTGGCATATCTCTCT |
| Ctgf | Forward | CCACCCGAGTTACCAATGAC |
| | Reverse | GCTTGGCGATTTTAGGTGTC |
| Fhl1 | Forward | TAGCTGTGCCCAGTCCTTCT |
| | Reverse | GCCATCCTTCTGCACGTACT |
| Fhl2 | Forward | AGGAGTGTGGAACACCCATC |
| | Reverse | AGCTGTTCCTCCTTGGCAG |
| Mkl1 | Forward | GATGCTAATGATGGCTGTGC |
| | Reverse | TCCAGGCTTCTTCTCTGCTC |
| Mkl2 | Forward | ATAGCTCCAAGAAGCAGCCA |
| | Reverse | TCCTTTGTTGCAGCCTCAG |
| Itgb1 | Forward | ATGCCAAATCTTGCGGAGAAT |
| | Reverse | TTTGCTGCGATTGGTGACATT |
| Rock1 | Forward | GTCGACTGGGGACAGTTTTG |
| | Reverse | AGGCAGGAAAATCCAAATCA |
| Rac1 | Forward | AGTGTGTGGTGGTGGGAGA |
| | Reverse | GCCCAGATTCACTGGTTTTC |
| Rac3 | Forward | CATCAAGTGCGTGGTGGTT |
| | Reverse | GGTTAACTGGCTTCCCATCC |
| RhoA | Forward | ACAACTGCATCCCAGAACCT |
| | Reverse | ACCTCTGGGAACTGGTCCTT |
| Cdc42 | Forward | GGGATCTGAAGGCTGTCAAG |
| | Reverse | ATGCGTTCATAGCAGCACAC |

## Slide 8
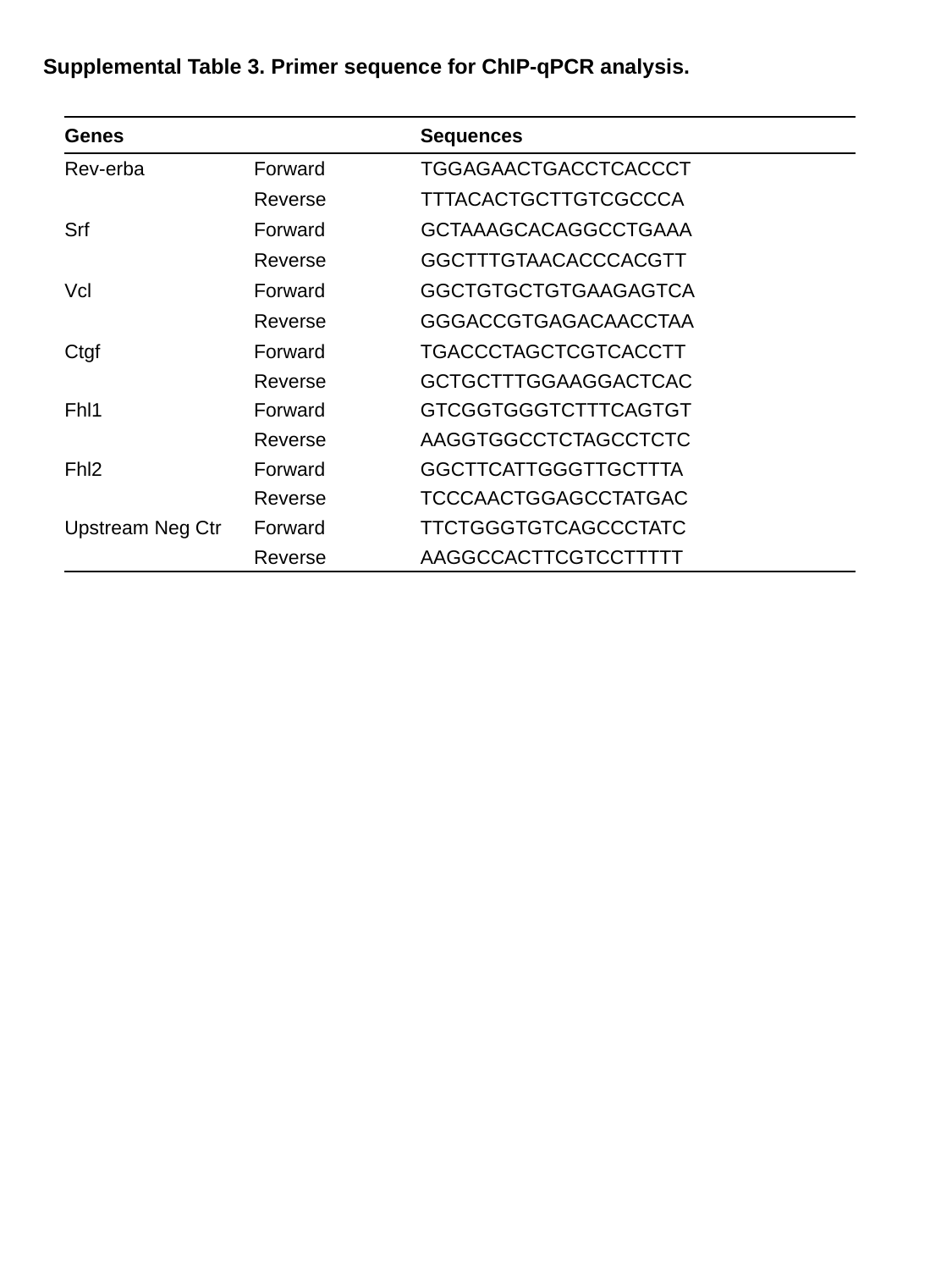

Supplemental Table 3. Primer sequence for ChIP-qPCR analysis.
| Genes | | Sequences |
| --- | --- | --- |
| Rev-erba | Forward | TGGAGAACTGACCTCACCCT |
| | Reverse | TTTACACTGCTTGTCGCCCA |
| Srf | Forward | GCTAAAGCACAGGCCTGAAA |
| | Reverse | GGCTTTGTAACACCCACGTT |
| Vcl | Forward | GGCTGTGCTGTGAAGAGTCA |
| | Reverse | GGGACCGTGAGACAACCTAA |
| Ctgf | Forward | TGACCCTAGCTCGTCACCTT |
| | Reverse | GCTGCTTTGGAAGGACTCAC |
| Fhl1 | Forward | GTCGGTGGGTCTTTCAGTGT |
| | Reverse | AAGGTGGCCTCTAGCCTCTC |
| Fhl2 | Forward | GGCTTCATTGGGTTGCTTTA |
| | Reverse | TCCCAACTGGAGCCTATGAC |
| Upstream Neg Ctr | Forward | TTCTGGGTGTCAGCCCTATC |
| | Reverse | AAGGCCACTTCGTCCTTTTT |

## Slide 9
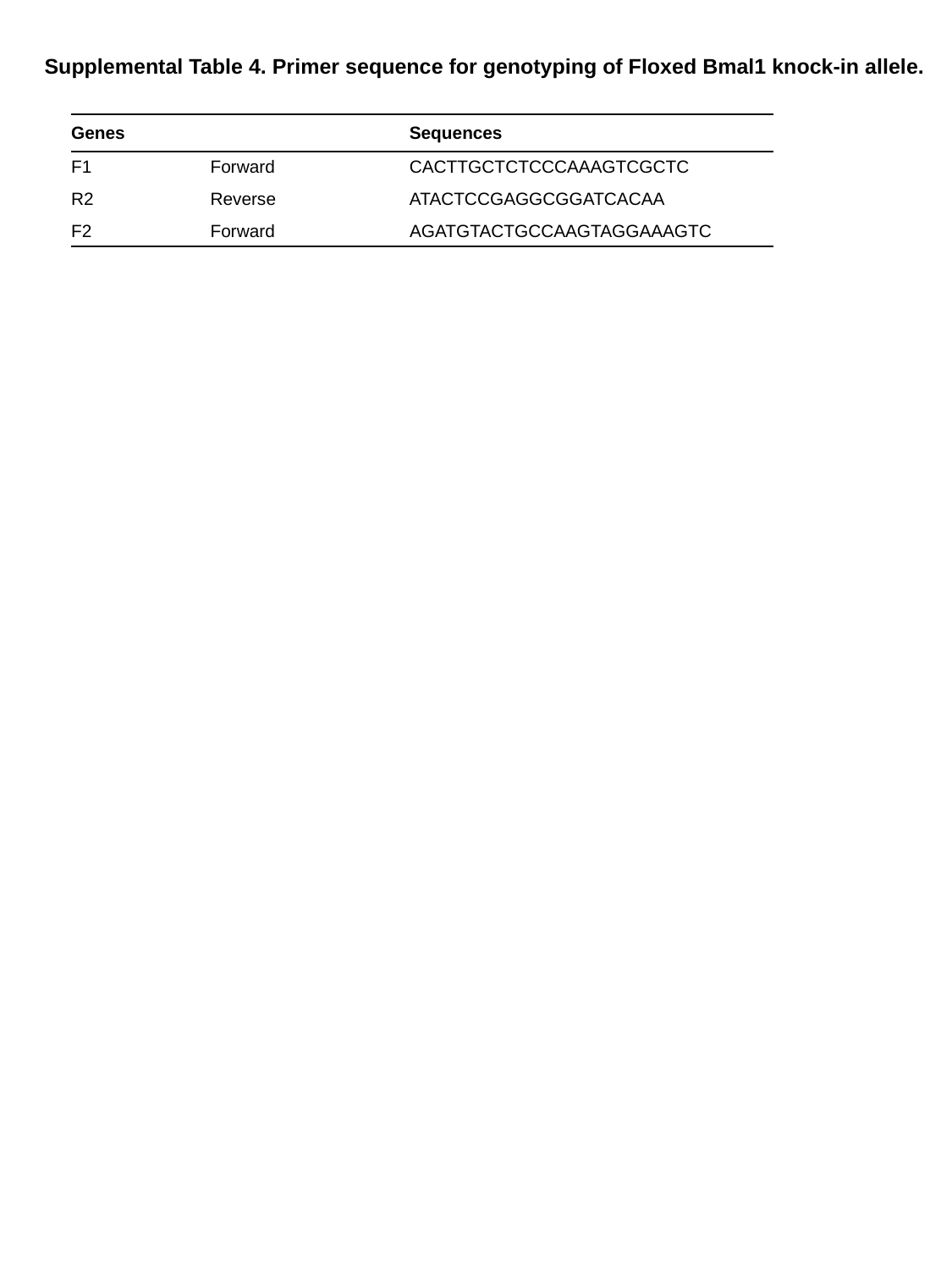

Supplemental Table 4. Primer sequence for genotyping of Floxed Bmal1 knock-in allele.
| Genes | | Sequences |
| --- | --- | --- |
| F1 | Forward | CACTTGCTCTCCCAAAGTCGCTC |
| R2 | Reverse | ATACTCCGAGGCGGATCACAA |
| F2 | Forward | AGATGTACTGCCAAGTAGGAAAGTC |
